# Systematic Review of Postvaccination Ocular Adverse Events: A Comprehensive Analysis of Published Reports

**DOI:** 10.1002/jmv.70747

**Published:** 2025-12-05

**Authors:** Yaru Zou, Koju Kamoi, Yuan Zong, Jing Zhang, Mingming Yang, Kyoko Ohno‐Matsui

**Affiliations:** ^1^ Department of Ophthalmology & Visual Science, Graduate School of Medical and Dental Sciences Institute of Science Tokyo Tokyo Japan

**Keywords:** antiviral vaccine, uveitis, vaccine adverse events, vaccine‐associated uveitis

## Abstract

Ocular adverse events following COVID‐19 vaccination are well described; however, systematic analyses of non‐COVID antiviral vaccines remain limited. This review aimed to evaluate ocular complications associated with non‐COVID antiviral immunizations, including influenza, varicella‐zoster (VZV), human papillomavirus (HPV), and hepatitis B (HBV) vaccines. A systematic search (PROSPERO CRD4202450873) identified 122 patients (184 eyes) from 8,487 publications, including case reports, case series, and observational studies. Uveitis was the most common (92/184 eyes; 50.0%, 95% CI 42.8%–57.2%), frequently following influenza vaccination (35/122; 28.7%, 95% CI 20.7%–36.7%). Most patients (95/122; 77.9%, 95% CI 70.5%–85.3%) required systemic corticosteroids, reflecting predominant inflammation. Ocular symptoms occurred within 30 days in 84.4% (103/122)of cases, with peak onset at 7–30 days (62/122; 50.8%, 95% CI 42.0%–59.6%). Despite appropriate treatment, 18 patients (20.0%, 95% CI 13.0%–29.4%) experienced persistent inflammation or required therapy beyond 1 month, categorized as “long‐vax”, defined as ocular symptoms persisting for ≥ 4 weeks after vaccination. Although rare, antiviral vaccine–associated ocular complications may persist, posing a risk of long‐term visual morbidity and emphasizing the importance of clinician awareness, postvaccination surveillance, and counseling for patients receiving repeated or combined vaccine exposures.

## 1 Introduction

The introduction of vaccines has been a pivotal advancement in the control and prevention of infectious diseases. From live attenuated and inactivated vaccines to recombinant DNA and mRNA platforms, immunization strategies have significantly reduced the global burden of viral illness [1]. Despite improved safety profiles, vaccines contain immunogenic components—such as antigens, adjuvants, and lipid carriers—that may trigger adverse immune responses in susceptible individuals [2].

Several mechanisms have been proposed to explain vaccine‐induced ocular inflammation [3]. These include molecular mimicry between viral and ocular antigens [4, 5], immune complex deposition within uveal or retinal vessels [6], delayed‐type hypersensitivity reactions [7], and adjuvant‐induced autoimmunity (ASIA) [8]. Such mechanisms may result in a spectrum of manifestations ranging from mild anterior uveitis to severe retinal necrosis or optic neuropathy.

As recurrent and emerging viral threats continue to challenge global health, there is increasing emphasis on identifying and characterizing potential adverse events following immunization (AEFI). Since 2000, the World Health Organization (WHO) has provided guidelines for the detection and classification of vaccine‐related adverse events in clinical trials [9]. According to Good Clinical Practice (GCP), an AEFI is defined as “any untoward medical occurrence following immunization,” regardless of causal relationship.

In this review, we stratified AEFIs based on the temporal persistence of ocular symptoms, defining “short‐vax” events as those resolving within 4 weeks, and “long‐vax” as those persisting beyond 4 weeks or resulting in permanent visual sequelae. This classification, informed by prior definitions of post‐vaccination inflammatory syndromes and long‐term immune activation [10], provides a clinically relevant framework to distinguish transient immune reactions from those with potential for chronic ocular damage.

While recent literature has focused extensively on COVID‐19‐related autoimmune phenomena, including rheumatic diseases [11], Behcet's syndrome [12], and musculoskeletal diseases [13], and ocular complications [14, 15, 16], systematic analyses of non‐COVID antiviral vaccines remain scarce. However, most existing literature comprises isolated case reports, and no systematic synthesis has been conducted to date. Given the expanding global use of antiviral vaccines, including seasonal influenza, herpes zoster, human papillomavirus, and other viruses [17, 18, 19, 20]. A consolidated review is urgently needed to inform clinicians and researchers.

This systematic review aims to fill this gap by compiling and analyzing published cases of ocular adverse events following vaccination against recurrent viral pathogens. Specifically, we aim to (1) describe the clinical characteristics and timing of reported ocular events, (2) identify patterns in causative vaccines, and (3) raise awareness among healthcare professionals regarding early detection and management of postvaccination ocular complications.

## 2 Methods

### 2.1 Data Sources and Search Strategy

The systematic review procedure was conducted according to the PRISMA guidelines [21]. The study protocol was published in the International Prospective Register of Systematic Reviews (CRD4202450873). A comprehensive search was conducted in PubMed, Embase, and the Cochrane Library databases for studies published between January 1, 2003 and October 1, 2023. The start date was selected to align with the WHO's publication of standardized post‐marketing vaccine safety surveillance guidelines in 2000, which improved the consistency and reliability of adverse event reporting.

In addition, the timeframe ensured inclusion of vaccines most relevant to contemporary practice, such as HPV, recombinant zoster, and mRNA‐based formulations, thereby enhancing applicability to current ophthalmic and immunologic contexts.

The search strategy incorporated combinations of keywords and Medical Subject Headings (MeSH) related to ocular conditions (e.g., “Eye Disease,” “eye disorders,”) and vaccine‐related terms (e.g., “vaccines,” “post‐vaccination,” “vaccine‐associated,” “autoimmunity”). Full search strings are detailed in Table S1. Reference lists of eligible studies were manually reviewed to identify additional relevant publications.

### 2.2 Inclusion and Exclusion Criteria

Studies were eligible for inclusion if they reported individual cases or case series of ocular adverse events temporally associated with vaccination against licensed antiviral pathogens (e.g., influenza, herpes zoster, HPV). Observational studies were included when sufficient individual‐level data were available to establish a temporal link between vaccination and ocular outcomes. The inclusion criteria were not intended to restrict study type but to ensure data completeness and clinical reliability. A summary of inclusion and exclusion criteria is provided in Table S2.

During the study selection process, studies were excluded at the title and abstract screening stage if they (1) did not report ocular disease, (2) were unrelated to Postvaccination or vaccine‐induced adverse reactions, (3) were nonhuman studies (in vitro, in vivo, or animal‐based), (4) were unrelated to antiviral vaccines, (5) involved multiple confounding factors other than vaccination, (6) were not available in full text, or (7) were publication types such as systematic reviews, meta‐analyses, scoping or narrative reviews, and commentaries. Detailed information on excluded studies is provided in Table S3. At the full‐text review stage, additional exclusions were applied for studies that could not be standardized, reported multiple syndromes or diseases combined, did not have ocular disease as the main manifestation, or contained implausible or insufficient individual data (detailed information is provided in Table S4).

### 2.3 Study Selection

**Figure 1:**
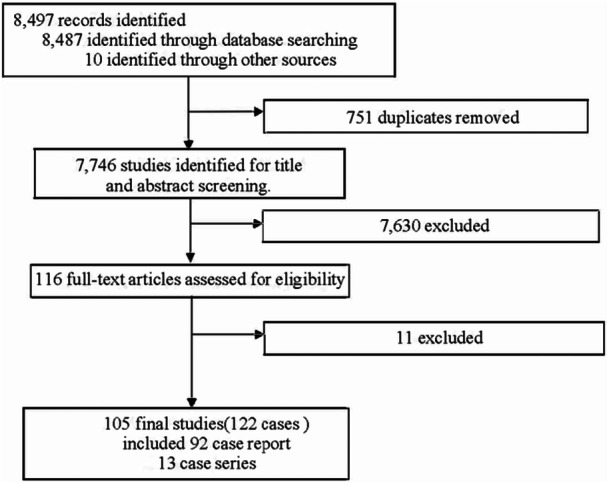
Flow chart of evaluated studies as per PRISMA guidelines.

Four reviewers (Yaun Zong, Yaru Zou, Mingming Yang, and Jing Zhang) independently screened titles and abstracts using predefined eligibility criteria, followed by full‐text review. All disagreements were resolved by consensus through discussion with a senior reviewer (Koju Kamoi). This process ensured high consistency and methodological rigor, in line with PRISMA recommendations for qualitative systematic reviews. Language was not an exclusion criterion, although all included texts were published in English. The study selection process is illustrated in the PRISMA flow diagram (Figure 1). A total of 105 studies were included: 92 case reports and 13 case series.

### 2.4 Data Extraction

From each included study, the following data were extracted using a standardized template:

Vaccine information**:** vaccine name, formulation (e.g., live attenuated, inactivated, recombinant, mRNA).

Patient demographics: age, gender.

Ocular diagnosis: type and laterality of ocular involvement.

Timing: interval between the most recent vaccination and onset of ocular symptoms.

Systemic symptoms and relevant comorbidities.

Clinical course: treatment administered (e.g., systemic corticosteroids, antivirals, topical therapy).

Outcomes: complete resolution, stability/recovering, and risk of long‐term side effects

Time intervals from vaccination to symptom onset were grouped into: ≤ 24 h, 24 h‐7 days, 7–30 days and > 30 days. Follow‐up durations were classified as < 1 month, ≥ 1 month. Ocular outcomes were categorized as:

Short‐vax: symptoms resolved within 4 weeks postvaccination.

Long‐vax: symptoms persisted ≥ 4 weeks or resulted in irreversible visual impairment.

All extracted data are summarized in Table S5.

### 2.5 Data Synthesis and Analysis

This review was primarily descriptive in nature. Continuous variables were summarized as means and standard deviations, and categorical variables as frequencies and percentages with 95% confidence intervals (CIs). Group comparisons were performed according to vaccine type, ocular diagnosis, symptom‐onset interval, treatment modality, and clinical outcome. Continuous variables were compared using the Mann–Whitney *U* test, and categorical variables using the χ² test or Fisher's exact test, as appropriate. To account for the within‐subject correlation between bilateral eyes, a generalized estimating equation (GEE) logistic regression model was applied to identify factors associated with long‐vax. Because the included studies consisted predominantly of heterogeneous case reports and small case series lacking standardized quantitative measures, a formal meta‐analysis was not feasible. Instead, data were synthesized descriptively in accordance with PRISMA recommendations for heterogeneous clinical evidence. All statistical analyses were performed using IBM SPSS Statistics for Windows, version 29.0 (IBM Corp., Armonk, NY, USA), and a two‐tailed *p* value < 0.05 was considered statistically significant.

## 3 Results

### 3.1 Overview of Included Studies and Cases

The database search retrieved 8487 articles, with 10 additional articles identified via manual citation search. In total, 105 studies that met the inclusion criteria, encompassing 122 patients and 184 affected eyes (Figure 1). Details of extracted data are presented in Table S5, and classification of 11 documented vaccine types is shown in Table S6.

Ocular events were defined as vaccine‐related if they occurred within one year of vaccination. Due to the latent potential of varicella‐zoster virus (VZV), two patients with onset at 3‐ and 8‐year postvaccination were also included. The most prevalent ocular adverse event was uveitis (*n* = 92/184, 50.0%, 95% CI 42.8%–57.2%), including anatomically classified uveitis (*n* = 26/184, 14.1%) (such as anterior, intermediate, posterior), panuveitis, and inflammation involving the uvea and other ocular structures (*n* = 66/184, 35.9%). Optic neuropathy was reported in 69 eyes (37.5%, 95% CI 30.4%–44.6%), with 38 eyes diagnosed with optic neuritis (20.7%); 18 eyes were associated with myelitis or encephalitis (9.8%). Other less frequently affected structures included the retina (11 eyes), cornea (7 eyes), and conjunctiva (5 eyes) (Table 1).

**Table 1:** Postvaccination ocular disease classification in all 105 studies (122 patients, 184 eyes), 2003–2023.

Ocular diseases	Eyes, *n* (%)	Interval range^a^	95% CI
Uveitis	92 (50.0)	24 h–8 years	42.8%–57.2%
Uveitis anatomically classified	26 (14.1)	24 h–10 weeks	
Diseases involving the uvea and other ocular structure	66 (35.9)	24 h–8 years	
ARN	13 (7.1)	2 days–16 months	
VKH	12 (6.5)	2 days–1 month	
APMPPE	9 (4.9)	10 days–3 weeks	
MEWDS	8 (4.3)	7 days–1 month	
Harada‐like disease	6 (3.3)	7 days–3 weeks	
Posterior uveitis and ERD	5 (2.7)	2–24 days	
MFC	3 (1.6)	24 h–3 weeks	
Keratouveitis	3 (1,6)	2 weeks–8 years	
Panuveitis with OIS	3 (1.6)	3–4 days	
AIBSES	2 (1.1)	11–20 days	
Retinal artery vasculitis with anterior uveitis	1 (0.5)	8 weeks	
Anterior uveitis with iris heterochromia and cataract	1 (0.5)	3 months	
Optic neuropathy	69 (37.5)	2 h–9 months	30.4%–44.6%
Optic neuritis	38 (20.7)	Few hours–25 days	
Optic neuritis with myelitis or encephalitis	18 (9.8)	2 days–6 months	
Nerve palsy	5 (2.7)	4 days–3 weeks	
Orbital myositis/Ptosis	5 (2.7)	2 h–2weeks	
Sequential Non‐Arteritis anterior ischemic optic neuropathy	2 (1.1)	1st:10 days; 2nd:6 days	
Optic neuropathy with optic disc edema	1 (0.5)	2 days	
Retinal disease	11 (6.0)	4 days–2 months	2.5%–9.4%
Corneal disease	7 (3.8)	1 week–1 year	1.0%–6.6%
Conjunctival disease	5 (2.7)	12 days–5 months	0.35%–5.10%
Total	184 (100)	2 h–8 years	

Abbriviations: AIBSES, acute idiopathic blind spot enlargement syndrome; APMPPE, acute posterior multifocal placoid pigment epitheliopathy; ARN, acute retinal necrosis; ERD, exudative retinal detachment; MEWDS, multiple evanescent white dot syndrome; MFC, multifocal choroiditis; OIS, orbital inflammatory syndrome; VKH, Vogt–Koyanagi–Harada.

^a^ Time between last vaccination and initial ocular symptom/sign.

### 3.2 Demographics and Clinical Features

As shown in Table 2, the cohort included 65 females (53.3%, 95% CI 44.5%–62.1%), with an average age of 37.4 years. Laterality of involvement was balanced (unilateral: bilateral = 60:62), with the left eye more commonly affected among unilateral cases (36/60, 60%).

**Table 2:** Patient characteristics (122 patients).

Variable	Total Sample (*N* = 122) (% values relate to the number of reports for each variable)	95% CI
Sex female, *n* (%)	65 (53.3)	44.5%–62.1%
Age, M (SD), range	37.4 (23.4)	33.2–41.6
Unilateral eye disease	60 (49.2)	40.3%–58.1%
Left	36 (60.0)	
Right	24 (40.0)	
Bilateral eye disease	62 (50.8)	41.9%–59.7%
Vaccine Generic		
Influenza vaccine, *n* (%)	35 (28.7)	20.7%–36.7%
Varicella zoster virus (VZV) vaccine, *n* (%)	29 (23.8)	16.3%–31.3%
Human papillomavirus (HPV) vaccine, *n* (%)	14 (11.5)	5.9%–17.1%
Measles‐Mumps‐Rubella (MMR) vaccine, *n* (%)	11 (9.0)	4.0%–14.0%
Combined use of multiple vaccines*	10 (8.2)	3.4%–13.0%
Yellow Fever virus vaccine, *n* (%)	8 (6.6)	2.2%–11.0%
Rabies vaccine, *n* (%)	6 (4.9)	1.1%–8.7%
Hepatitis B virus (HBV) vaccine (%)	4 (3.3)	0.1%–6.5%
Hepatitis A virus (HAV) vaccine, *n* (%)	2 (1.6)	0%–3.7%
Monkeypox virus, *n* (%)	1 (0.8)	0%–2.4%
Smallpox virus, *n* (%)	1 (0.8)	0%–2.4%
Poliovirus vaccine, *n* (%)	1 (0.8)	0%–2.4%
Intervals		
≤ 24 h	9 (7.4)	2.7%–12.1%
24 h–7 days	32 (26.2)	18.5%–33.9%
7 days–30 days	62 (50.8)	42.0%–59.6%
> 30 days	18 (14.8)	8.5%–21.1%
Not reported	1 (0.8)	0–2.4%
Systemic symptoms		
With, *n* (%)	61 (50.0)	41.1%–58.9%
Without, *n* (%)	52 (42.6)	
Not reported, *n* (%)	9 (7.4)	
Treatment		
Systemic steroids, *n* (%)	95 (77.9)	70.5%–85.3%
Not reported	3 (2.5)	
No treatment, *n* (%)	24 (19.7)	
Follow‐up period		
≥ 1 month, *n* (%)	83 (68.0)	59.7%–76.3%
< 1 month, *n* (%)	7 (5.7)	1.6%–9.8%
Not reported, *n* (%)	32 (26.2)	
Prognosis		
Complete recovery, *n* (%)	56 (45.9)	37.1%–54.7%
Stability/recovering, *n* (%)	37 (30.3)	22.1%–38.5%
Risk of long‐term side effects, *n* (%)	21 (17.2)	10.4%–24.0%
Not reported, *n* (%)	8 (6.6)	

*Combined use of multiple vaccines: Yellow Fever + HAV+meningitis vaccine; HAV+typhoid+yellow fever vaccine; HPV+Meningococcus vaccine; HAV+polio,+tetanus+meningococcal vaccine; HBV+Tdap vaccine; Haemophilus influenzae type b+Pneumococcal conjugate + MMR vaccine; Yellow Fever+meningitis vaccine; diphtheria+tetanus+polio + HAV+typhoid vaccine.

Systemic symptoms were reported in half of the patients (61/122, 50.0%, 95% CI 41.1%–58.9%). The most frequently implicated vaccine was influenza (35/122, 28.7%, 95% CI 20.7%–36.7%), followed by VZV (29/122, 23.8%, 95% CI 16.3%–31.3%), Human Papillomavirus (HPV) (14/122, 11.5%, 95% CI 5.9%–17.1%), and Measles‐Mumps‐Rubella (MMR) (11/122, 9.0%, 95% CI 4.0%–14.0%). The time interval between vaccination and the onset of initial ocular manifestations ranged from 24 h to 8 years. Most patients developed ocular symptoms between 7 and 30 days postvaccination (*n* = 62, 50.8%, 95% CI 42.0%–59.6%).

Regarding treatment, 24 patients did not receive any treatment. Most patients (95/122, 77.9%, 95% CI 70.5%–85.3%) received systemic steroid therapy. Most cases explicitly mentioned the follow‐up period, with 83/122 (68.0%, 95% CI 59.70%–76.3%) patients having follow‐up periods of ≥ 1 month. The prognosis was categorized into three types: 56/122 (45.9%, 95% CI 37.1%–54.7%) patients recovered completely, 37/122 (30.3%, 95% CI 22.1%–38.5%) patients had stable conditions or significant improvement from baseline, and 21/122 (17.2%, 95% CI 10.4%–24.0%) patients faced long‐term risk.

### 3.3 Long‐Vax and Short‐Vax

**Figure 2:**
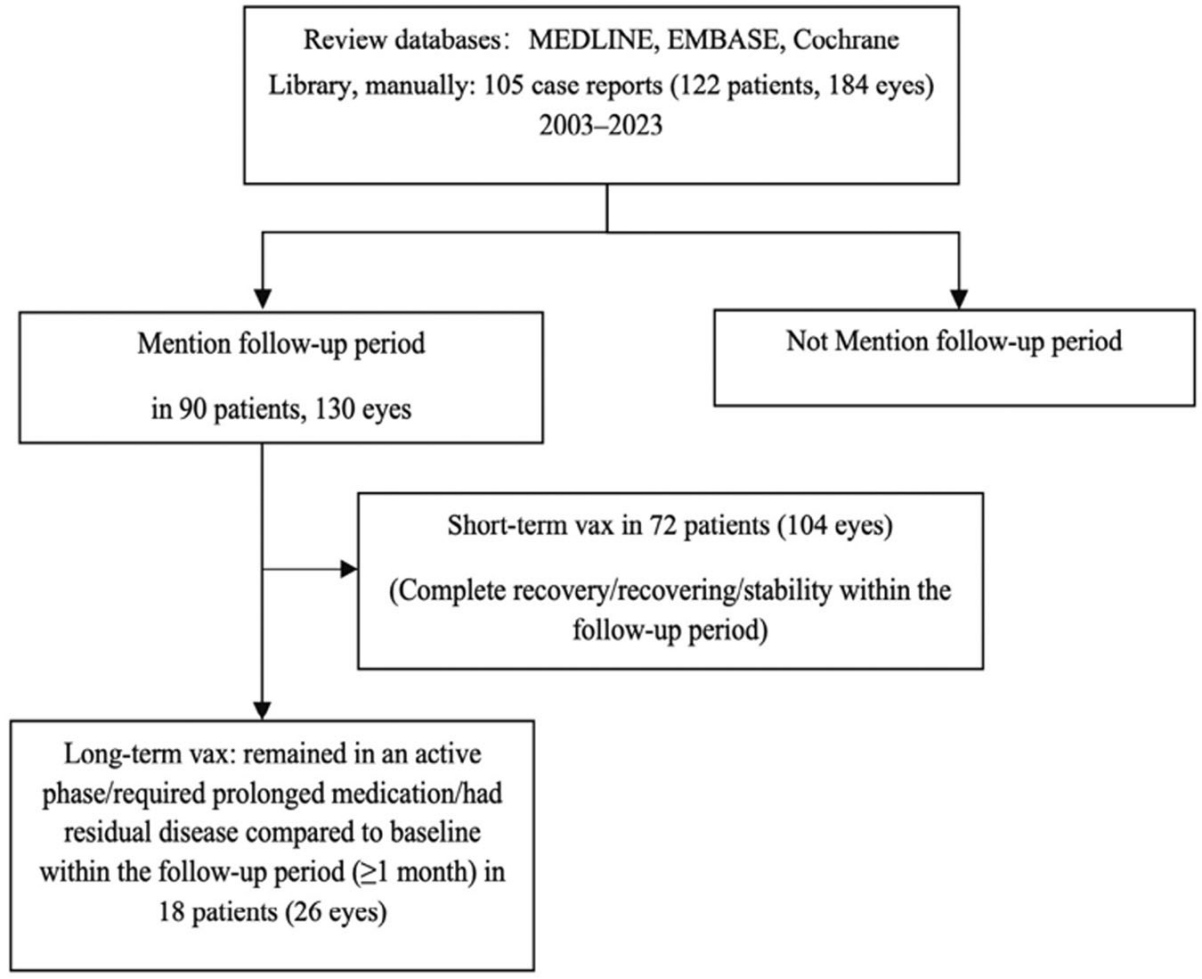
Follow‐up outcomes following vaccination among case reports.

To evaluate long‐term versus short‐term ocular outcomes following vaccination, we analyzed 90 cases with documented follow‐up periods. Among them, 72 patients experienced complete resolution, ongoing recovery, or disease stabilization within 1 month and were categorized as “short‐vax,” while 18 patients continued to exhibit symptoms, required extended treatment, or had residual visual dysfunction after at least 1 month and were classified as “long‐vax.” These 18 patients accounted for 26 affected eyes, with two patients showing improvement in one eye while the other remained unchanged. The overall selection process and classification are illustrated in Figure 2 and detailed in Table S7.

### 3.4 Association Between Long‐**Vax** and Relevant Variables

To ensure accurate temporal interpretation, two patients with delayed onset of ocular symptoms (more than 3 years after vaccination) were excluded, resulting in a final cohort of 88 patients (128 eyes), as summarized in Table S7. The mean age was comparable between the long‐vax (35.20 ± 24.28 years) and short‐vax (35.43 ± 21.48 years) groups. The most frequently implicated vaccines were influenza, VZV, and HPV vaccines, as well as the combined use of multiple vaccines, although no vaccine type showed a significant association with long‐vax in the GEE analysis (Table S8). Uveitis was the most common ocular manifestation, observed in 50.0% (13/26) of long‐vax and 52.0% (53/102) of short‐vax eyes, followed by optic neuropathy (26.9% vs 37.3%) and retinal disease (19.2% vs 5.9%), none of which were significantly associated with long‐vax (*p* > 0.05 in GEE models). The onset pattern differed modestly between groups: in the long‐vax group, half of the eyes (13/26, 50.0%) developed symptoms within 7 days postvaccination, whereas in the short‐vax group, most cases (58/102, 56.9%) manifested between 7 and 30 days, yet the interval was not statistically significant (7–30 days: B = −0.713, 95% CI − 1.799 to 0.374, *p* = 0.198; > 30 days: *B* = −1.106, 95% CI −3.324 to 1.112, *p* = 0.328) (Table S8). Systemic symptoms were relatively common in both groups (69.2% vs. 58.8%). Importantly, systemic corticosteroid use was the only variable significantly associated with long‐vax (*β* = 2.192, 95% CI 0.117–4.267; *p* = 0.038), with 96.2% (25/26) of long‐vax and 69.6% (71/102) of short‐vax patients receiving systemic steroids. This finding likely reflects a clinical correlation between systemic inflammation and delayed onset, rather than a direct causal relationship.

Table S9 summarizes the clinical characteristics of 26 eyes with long‐vax. The most frequently involved vaccines were HPV, influenza, VZV, and the combined use of multiple vaccines, each accounting for 19.2% of cases (5 eyes each), followed by yellow fever (15.4%, 4 eyes) and hepatitis B (7.7%, 2 eyes). Regarding ocular manifestations, uveitis was the predominant diagnosis, affecting 13 eyes (50.0%), while optic neuropathy was observed in 7 eyes (26.9%), and retinal disease in 5 eyes (19.2%). One eye (3.8%) presented with corneal disease. Anatomically, uveitis mainly included multifocal choroiditis (MFC, *n* = 2), posterior uveitis with exudative retinal detachment (ERD, *n* = 2), Vogt–Koyanagi–Harada disease (VKH, *n* = 2), and other entities such as acute idiopathic blind spot enlargement syndrome (AIBSES), acute retinal necrosis (ARN), multiple evanescent white dot syndrome (MEWDS), and panuveitis with orbital inflammatory syndrome (OIS). Functionally, disturbance of visual acuity was noted in 9 eyes (34.6%), long‐term drug therapy was required in 8 eyes (30.8%), and visual‐field defects, persistent vascular leakage, and uveitis with diffuse retinal vasculitis were each reported in 3 eyes (11.5%). These findings indicate that long‐vax eyes were frequently characterized by chronic inflammation requiring prolonged treatment and sometimes accompanied by vascular or structural complications.

## 4 Discussion

### 4.1 Ocular Disease

This review compiles case reports and a series of ocular side effects following mainstream antiviral vaccine administration over the past 20 years. Such a systematic synthesis allows not only the identification of frequently reported entities but also provides insight into patterns of disease severity and prognosis. Consistent with our previous study [22] and recent reviews spanning from 2010 to 2020 [1], uveitis emerged as the most frequently reported ocular adverse event (92/184, 50%, 95% CI 42.8%–57.2%), followed by optic neuropathy—primarily optic neuritis—and retinal diseases.

Uveitis, defined as inflammation of the uveal tract [23], was further classified anatomically (26/184, 14.1%) or as involving multiple ocular structures (66/184, 35.9%). Wong et al. have categorized entities such as MFC, AIBSES, MEWDS, and acute posterior multifocal placoid pigment epitheliopathy (APMPPE) as primary inflammatory choriocapillaropathies (PICCP), typically with favorable prognoses [24]. However, our cohort also included more severe manifestations—such as keratouveitis, posterior uveitis with ERD, panuveitis with OIS, ARN, VKH disease, and persistent MEWDS, AIBSES, and MFC—many of which were associated with prolonged symptoms (Table 1). Notably, 7 out of 13 patients with these conditions (53.8%) experienced significant visual impairment and required long‐term corticosteroid therapy. This finding underscores that, although many postvaccination uveitic entities are self‐limited, a substantial subset progresses to vision‐threatening disease necessitating intensive and extended treatment.

Optic neuropathy, particularly optic neuritis and neuromyelitis optica (NMO), was the second most common postvaccination ocular condition (69/184, 37.5%). Prior studies have linked influenza and HPV vaccines to demyelinating diseases. Kumar et al. found a strong association between influenza and HPV vaccination with demyelinating diseases [25], while Stübgen et al. observed onset typically within 3 weeks of vaccination [26]. In our analysis, symptom onset ranged from a few hours to 25 days (Table 1). While most cases responded well to treatment, a subset experienced severe or permanent vision loss (Table S9). These findings align with the hypothesis that vaccine‐induced demyelinating events may be immune‐mediated and heterogenous in severity, with some patients experiencing irreversible axonal damage.

Among seven patients (11 eyes) with retinal disease, yellow fever vaccination was frequently associated with long‐vax presentations, including recurrent vascular leakage, retinal vasculitis, and progressive atrophy. These outcomes may be attributable to transient viremia and potential neuroinvasion linked to the vaccine's derivation from the wild‐type Asibi strain 17D [27]. Given the poor outcomes observed in these cases, yellow fever–associated retinal involvement warrants clinical vigilance.

All seven patients diagnosed with corneal disease developed unilateral recurrent keratitis after VZV vaccination. Notably, one had a history of herpes zoster keratitis, and one tested positive for VZV. Grillo and colleagues reported 24 cases of VZV vaccine‐related keratitis [28]. Given these risks, screening and delaying vaccination for individuals with a relevant ocular or infectious history may be prudent. Although most patients improved with antiviral and steroid therapy, one required prolonged treatment [29]. This suggests that prior ocular herpes infection represents a potential risk factor for postvaccination keratitis recurrence and may inform individualized vaccination strategies.

### 4.2 Vaccine‐Specific Patterns

During  the 2019–2020 flu season, vaccination prevented an estimated 7 million influenza cases [30]. However, influenza vaccines also accounted for the highest proportion of postvaccination ocular side effects in our study (35/122, 28.7%), which may reflect their widespread administration. They were also linked to both the highest number of long‐term (5/26, 19.2%) and short‐term (36/102, 35.3%) ocular complications. Potential mechanisms include molecular mimicry, adjuvant‐induced autoimmune/inflammatory syndrome (ASIA), and incomplete viral inactivation [31]. While the H1N1 vaccine is adjuvant‐free and contains only trace amounts of thiomersal [32], adverse events associated with thiomersal, especially in pediatric populations, have been reported. The CDC has documented 88 cases of Kawasaki disease postvaccination since 1990 [33]. Taken together, these findings suggest that although influenza vaccines remain highly effective in preventing systemic morbidity, their immunological effects may unmask or exacerbate latent autoimmune ocular susceptibility in a subset of patients.

Our findings also suggest an association between VZV vaccination and ARN, particularly in elderly or immunocompromised individuals (Table S5). Although the herpes zoster vaccine boosts pre‐existing immunity to VZV [34], post‐immunization complications have been observed [35]. Among the long‐vax cases linked to VZV, most required extended or even lifelong treatment (Table S9). For immunocompromised patients, inactivated vaccines may present a safer option, although their overall risk‐benefit profile requires further clarification [36]. The possibility of latent virus reactivation underscores the importance of careful ophthalmic monitoring and individualized vaccine selection in high‐risk groups.

Regarding HPV vaccines, a safety evaluation published in JAMA reported 772 adverse events (6.2%) among recipients, most commonly syncope, dizziness, nausea, and headache [37]. Currently, limited clinical evidence supports a direct causal relationship between HPV vaccines and ocular disease. Nonetheless, in our study, most HPV‐associated ocular adverse events occurred in young women, likely reflecting targeted immunization policies [38]. Among the 17 affected eyes, 5 met the criteria for long‐vax, including persistent vascular leakage and visual impairment (Table S9). Although causality remains unproven, the clustering of demyelinating or vasculitic events after HPV vaccination warrants ongoing pharmacovigilance and mechanistic investigation.

### 4.3 Analysis of Long‐ and Short‐Vax

We adopted the previously reported definitions of long‐ and short‐vax [9], as used in post‐COVID‐19 vaccine adverse event studies [39, 40, 41]. Analysis of our cohort revealed associations between long‐vax outcomes and variables such as systemic corticosteroid use, interval to symptom onset, retinal involvement, and administration of multiple vaccines. These findings provide an early framework to stratify patients at risk of persistent postvaccination ocular disease.

### 4.4 Systemic Steroid Use

Of 122 patients, 95 received systemic steroids, which prevented permanent vision impairment [42]. However, some patients did not show significant improvement over 1 month. The observed association between systemic steroid use and prolonged ocular effects (*β* = 2.192, 95% CI 0.117–4.267; *p* = 0.038) remains noteworthy, despite not implying causality. (Table S8) Possible explanations include: (1) Steroid hesitancy: immunosuppressive effects of steroids increase viral susceptibility and cause ocular side effects such as elevated intraocular pressure (IOP), optic atrophy, and retinal embolism [43]. (2) Delayed steroid administration: In several cases, antiviral therapy was used first, postponing steroid administration. For example, one patient with ARN after herpes zoster vaccination experienced vision loss to light perception despite high‐dose combined therapy [44]. (3) Pre‐existing conditions: older individuals, infants, and those with underlying diseases like diabetes and immunosuppressant use were more prone to long‐vax [45]. Among 18 patients with long‐term side effects in our study, 3 had underlying diabetes, 1 used immunosuppressants, and 7 were children (Table S5). It is also important to acknowledge potential treatment‐reporting bias, as untreated or mild self‐limited cases are less likely to be published, potentially overestimating steroid use and underrepresenting benign outcomes.

### 4.5 Intervals

Most patients (*n* = 62/122, 50.8%) experienced ocular symptoms within 7–30 days postvaccination (Table 1). Long‐vax cases mostly emerged within the first 7 days (*n* = 13/26, 50.0%), whereas short‐vax cases occurred within 7–30 days (*n* = 58/102, 56.9%). These findings suggest that earlier onset of symptoms may be associated with a prolonged disease course. One possible explanation is that rapid onset reflects a heightened or dysregulated immune response to vaccination, which may increase the risk of sustained inflammation or immune‐mediated damage. Conversely, delayed onset within the 7–30‐day window likely corresponds to adaptive immune maturation [46]; a longer interval may support the development of antigen‐specific memory B cells and T cells [47], reduce antigenic competition in germinal centers, and promote a more balanced immune reaction [48]. This temporal distinction could therefore serve as a clinical predictor, guiding closer surveillance for patients who present with ocular events within the first week after vaccination. This temporal pattern also aligns with findings from other vaccine studies, including COVID‐19, where both reactogenicity and efficacy vary with dosing intervals [49]. Integrating these observations, it is plausible that ocular events occurring in the immediate post‐vaccine period may reflect maladaptive immune activation, whereas later onset could indicate more physiologic immune priming. This temporal distinction may help guide vaccine scheduling decisions and the intensity of ophthalmologic follow‐up.

### 4.6 Clinical Implications

Taken together, our findings underscore the need for individualized postvaccination monitoring. Clinicians should maintain a high index of suspicion for ocular symptoms occurring within the first week after antiviral vaccination, as these may herald prolonged inflammation. High‐risk populations, such as those with prior ocular herpes infection, autoimmune disorders, or on immunosuppressive therapy, may benefit from closer ophthalmic follow‐up within the first month postvaccination. Incorporating timely ophthalmologic evaluation and multidisciplinary coordination (e.g., with immunologists or infectious disease specialists) could help prevent irreversible visual loss. Future research should aim to establish standardized follow‐up intervals, identify immunogenetic risk factors, and evaluate prophylactic or early immunomodulatory strategies for susceptible individuals.

### 4.7 Limitations

Despite these findings, our study had certain limitations.

1. Temporal scope restriction: Limited to the past two decades, our analysis may not fully update in vaccine formulations or variations in quality among companies.

2. Publication bias of a single case report: Given the difficulty of establishing causality on an individual basis, journals tend to publish severely impactful case reports, leading to underreporting of similar but less severe cases, which may introduce bias.

3. Under‐consideration of underlying diseases: while discussed, underlying diseases were not included in the multifactorial analysis, potentially confounding the results.

4. Limitations of single‐case reports: Despite a comprehensive review, single‐case reports and case series cannot establish causality. More double‐blind randomized controlled trials and mechanistic studies are needed.

## 5 Conclusions

This study represents the first systematic review to comprehensively evaluate ocular adverse events following antiviral vaccination and to analyze their temporal characteristics in relation to long‐vax and short‐vax outcomes. Among the identified vaccines, influenza, VZV, and HPV vaccines were most frequently associated with ocular events. However, this predominance likely reflects their widespread global use and extensive post‐marketing surveillance, rather than an inherently higher risk of ocular complications. Retinal disease after yellow fever virus vaccination had a poor prognosis, and all ARN cases were linked to VZV vaccination. Uveitis and optic neuropathy were the most common complications, with onset intervals between 7 and 30 days. Longer intervals may reduce long‐term side effects risks. Most patients responded favorably to systemic corticosteroid therapy, although a subset experienced incomplete recovery. Given the retrospective and heterogeneous nature of available evidence, the apparent benefit of corticosteroids should be interpreted with caution; prospective studies are needed to confirm optimal timing, dosage, and duration of treatment.

Although ocular side effects remain rare and the overall benefits of vaccination far outweigh the risks, heightened clinical vigilance is warranted. Early recognition and timely steroid intervention are essential to preventing irreversible visual damage, especially in patients with systemic symptoms or underlying immune compromise.

## Author Contributions

Conceptualization, study design, Koju Kamoi, literature search, figures, data analysis, writing – original draft preparation, Yaru Zou; data interpretation, Koju Kamoi, Yaru Zou, Yuan Zong; writing – review and editing, data collection, Koju Kamoi, Yuan Zong, Jing Zhang, Mingmong Yang, and Kyoko Ohno‐Matsui; funding acquisition, Koju Kamoi and Yaru Zou. All authors have read and agreed to the published version of the manuscript.

## Ethics Statement

No human subjects were included in this study. All research adhered to the tenets of the Declaration of Helsinki. This is a retrospective study using deidentified subject details. This study was exempt from IRB review. No animal subjects were included in this study.

## Consent

The requirement for informed consent was waived because of the retrospective nature of the study.

## Conflicts of Interest

All authors have completed and submitted the ICMJE disclosures form. The author(s) have no proprietary or commercial interest in any materials discussed in this article.

## Supporting information


**Supplementary Table 1:** Search Strategy. **Supplementary Table 2:** Selection criteria. **Supplementary Table 3:** Duplication Details of articles excluded after careful screening of titles and abstracts. **Supplementary Table 4:** Studies excluded post‐full text screening. **Supplementary Table 5:** The summary of basic information of all 105 studies (122 patients), 2003‐2023. **Supplementary Table 6:** Vaccines Potentially Implicated in Various Ocular Complications. **Supplementary Table 7:** Characteristics of long‐term side effects and variables (88 patients, 128 eyes). **Supplementary Table 8:** Factors associated with Long‐vax among 128 eyes. **Supplementary Table 9:** Description of 26 eyes in various vaccinations and types of Long‐vax.

## Data Availability

The data that support the findings of this study are available in the Supporting Information of this article. Data available in web‐only Supporting Material.
