# Supplementary material for: Systematic Review of Postvaccination Ocular Adverse Events: A Comprehensive Analysis of Published Reports
Source: J Med Virol. 2025 Dec 5;97(12):e70747. doi: 10.1002/jmv.70747 (PMC12679683; doi:10.1002/jmv.70747)
Supplement: Supplementary file 1 — Supplementary Table 1: Search Strategy. Supplementary Table 2: Selection criteria. Supplementary Table 3: Duplication Details of articles excluded after careful screening of titles and abstracts. Supplementary Table 4: Studies excluded post‐full text screening. Supplementary Table 5: The summary of basic information of all 105 studies (122 patients), 2003‐2023. Supplementary Table 6: Vaccines Potentially Implicated in Various Ocular Complications. Supplementary Table 7: Characteristics of long‐term side effects and variables (88 patients, 128 eyes). Supplementary Table 8: Factors associated with Long‐vax among 128 eyes. Supplementary Table 9: Description of 26 eyes in various vaccinations and types of Long‐vax. [file JMV-97-e70747-s001.docx]

**Online Supplement to**

**Systematic Review of Postvaccination Ocular Adverse Events: A Comprehensive Analysis of Published Reports**

**Supplementary Table 1: Search Strategy**

Supplementary Table 2. Selection criteria

Supplementary Table 3. Duplication Details of articles excluded after careful screening of titles and abstracts

Supplementary Table 4. Studies excluded post-full text screening

Supplementary Table 5: The summary of basic information of all 105 studies (122 patients), 2003-2023

**Supplementary Table 6: Vaccines Potentially Implicated in Various Ocular Complications**

Supplementary Table 7: Characteristics of long-term side effects and variables (88 patients, 128 eyes)

**Supplementary Table 8. Factors associated with Long-vax among 128 eyes.**

**Supplementary Table 9: Description of 26 eyes in various vaccinations and types of Long-vax**

**Abbreviations and Acronyms:**

Ab antibody

ADEM Acute Disseminated Encephalo-Myelitis

AEFI adverse events following immunization

AIBSES acute idiopathic blind spot enlargement syndrome

APMPPE acute posterior multifocal placoid pigment epitheliopathy

ARN acute retinal necrosis

ASIA adjuvant-induced autoimmune syndrome

DTP diphtheria, tetanus and polio

ERD exudative retinal detachment

ESR erythrocyte sedimentation rate

FA fluorescein angiography

gtt eyedrops

HAV hepatitis A virus

HBV hepatitis B virus

HLA human leukocyte antigen

HPV human papillomavirus

HZO herpes zoster ophthalmic

io intra-orbital/retrobulbar

iv intravenous

Long-vax long-term vaccine-related side effects

MEWDS multiple evanescent white dot syndrome

MFC multifocal choroiditis

MMR measles–mumps–rubella

NMO neuromyelitis optica

NMOSD neuromyelitis optica spectrum disorder

NR not reported

NSAIDs non-steroid anti-inflammatory drugs

OD right eye

OIS orbital inflammatory syndrome

OPV oral poliovirus type 2 vaccine

OS left eye

PAMM paracentral acute middle maculopathy

PICCP primary inflammatory choriocapillarisopathy

po orally

RPE retinal pigment epithelium

Short-vax short-term vaccine-related side effects

TINU tubulointerstitial nephritis and uveitis

VKH Vogt–Koyanagi–Harada

VZV varicella zoster virus

**Supplementary Table 1: Search Strategy**

| Database | Number | Search Strategy |
| --- | --- | --- |
| Pubmed | N=2405 | (("eye diseases"[MeSH Terms] OR "eye disorders"[Title/Abstract] OR "eye disorder"[Title/Abstract]) AND ("vaccines"[MeSH Terms] OR "vaccine"[Title/Abstract] OR "vaccine-associated"[Title/Abstract] OR "postvaccination"[Title/Abstract] OR "immunization"[Title/Abstract] OR "autoimmunity"[Title/Abstract] OR "hepatitis a vaccines"[Title/Abstract] OR "hepatitis b vaccines"[Title/Abstract] OR "human papillomavirus vaccines"[Title/Abstract] OR "herpes vaccines"[Title/Abstract] OR "influenza vaccines"[Title/Abstract] OR "measles vaccines"[Title/Abstract] OR "measles mumps rubella vaccines"[Title/Abstract] OR "smallpox vaccines"[Title/Abstract] OR "varicella vaccines"[Title/Abstract] OR "yellow fever vaccines"[Title/Abstract] OR "zoster vaccines"[Title/Abstract])) AND ((humans[Filter]) AND (2003:2023[pdat])) |
| Cochrane | N=68 | #1: MeSH descriptor: [Eye Diseases] this term only  #2:(Eye disease):ti,ab,kw OR (Eye Disorder):ti,ab,kw OR (Eye Disorders):ti,ab,kw  #3: #1 OR #2  #4: MeSH descriptor: [Vaccines] this term only  #5:(vaccine):ti,ab,kw OR (vaccine-associated):ti,ab,kw OR (postvaccination):ti,ab,kw OR (immunization):ti,ab,kw OR (autoimmunity):ti,ab,kw OR (hepatitis A vaccines):ti,ab,kw OR (hepatitis B vaccines):ti,ab,kw OR (Human papillomavirus vaccines):ti,ab,kw OR (herpes vaccines):ti,ab,kw OR (influenza vaccines):ti,ab,kw OR (measles vaccines):ti,ab,kw OR (Measles-Mumps-Rubella vaccines):ti,ab,kw OR (smallpox vaccines):ti,ab,kw OR (varicella vaccines):ti,ab,kw OR (yellow fever vaccines):ti,ab,kw OR (zoster vaccines):ti,ab,kw  #6: #4 OR #5  #7: #3 AND #6 |
| Embase | N=6014 | #1: 'vaccine'/exp OR vaccines:ab,ti OR 'vaccine associated':ab,ti OR 'postvaccination':ab,ti OR immunization:ab,ti OR autoimmunity:ab,ti OR 'hepatitis a vaccine':ab,ti OR 'hepatitis b vaccine':ab,ti OR 'human papilloma virus vaccine':ab,ti OR 'herpes vaccine':ab,ti OR 'influenza vaccine':ab,ti OR 'measles vaccine':ab,ti OR 'measles mumps rubella vaccine':ab,ti OR 'smallpox vaccine':ab,ti OR 'yellow fever vaccine':ab,ti OR 'varicella zoster vaccine':ab,ti  #2: 'eye diseases'/exp OR 'eye diseases':ab,ti OR 'eye disorder':ab,ti OR 'eye disorders':ab,ti  #3: #1 AND #2  #4: #1 AND #2 AND [humans]/lim AND [clinical study]/lim AND [2003-2023]/py |

**Note:** This study was conducted in 3 searches: PubMed, Embase, and Cochrane library. The following keywords were used for the search from Jan 1, 2003 to Oct 1, 2023, without language restrictions with the following search terms: “Eye Diseases”, “Eye disease”, “Eye Disorders”, “Eye Disorder” “Vaccines”, “Vaccine”, “vaccine-associated”, “postvaccination”, “immunization”, “autoimmunity”, “hepatitis a vaccines”, “hepatitis b vaccines”, “human papillomavirus vaccines”, “herpes vaccines”, “influenza vaccines”, “measles vaccines”, “measles mumps rubella vaccines”, “smallpox vaccines”, “varicella vaccines”, “yellow fever vaccines”, “zoster vaccines”, filtered by humans. The original databases search retrieved 8487 articles, and citation searching led to the identification of an additional 10 articles.

Supplementary Table 2. Selection criteria

| Principle | Inclusion Criteria | Exclusion Criteria |
| --- | --- | --- |
| Participants | Studies providing detailed description of cases of postvaccination ocular diseases | In vitro, in vivo, epidemiological studies, or reviews on postvaccination ocular diseases, Implausible/ insufficient individual data. |
| Intervention | Any licensed anti-viral vaccines | Unlicensed vaccines, vaccines against bacteria are not within the scope of our discussion |
| Outcome | Eye disease is the main clinical manifestation of the patient, more comprehensive personal information | Syndrome or multiple diseases combined; eye disease is not its main manifestation; unable to standardize. |
| Study design | Cohort studies, case reports, and series were eligible for inclusion | Studies providing aggregate data on postvaccination ocular diseases, without detailed descriptions of individual cases |

Supplementary Table 3. Duplication Details of articles excluded after careful screening of titles and abstracts

| **Type** | **Reasons for exclusion** | **Number of studies** |
| --- | --- | --- |
| Duplicate Studies | Software | 573 |
|  | manually | 178 |
| Studies excluded during screening | Not eye disease | 4558 |
|  | Not a postvaccination/vaccine-induced adverse reaction | 1011 |
|  | Not human (in vitro, in vivo, animal-based study) | 243 |
|  | Not related vaccine (not anti-viral vaccine) | 1741 |
|  | Vaccines are not the only influencing factor | 10 |
|  | Full article not found | 4 |
|  | systematic reviews/meta-analysis, scoping, review/narrative review, commentary | 63 |

Supplementary Table 4. Studies excluded post-full text screening

| **Type** | **Study** | **Reasons for exclusion** |
| --- | --- | --- |
| Unable to standardize | Ho DK, Stevenson SR, Kumar V. Bilateral Cystoid Macular Edema and Corneal Endothelial Graft Rejection following Influenza and Varicella-Zoster Vaccinations. Ocular immunology and inflammation 2023; 31(3): 656-9. | The patient developed symptoms in his left eye 8 weeks after receiving the influenza vaccine, and symptoms in his right eye four weeks later, which improved within 4 months. He then received the VZV vaccine, and ocular symptoms appeared again 11 days later. Due to the complex course of the disease and the inconsistent onset of symptoms in both eyes, it was not possible to standardize, and the final decision was not to include them. |
| Syndrome or multiple diseases combined; eye disease is not its main manifestation | Nichani P, Micieli JA. Granuloma Annulare, Scalp Necrosis, and Ischemic Optic Neuropathy from Giant Cell Arteritis After Varicella-Zoster Virus Vaccination. Journal of neuro-ophthalmology: the official journal of the North American Neuro-Ophthalmology Society 2021; 41(2): e145-e8. | After VZV vaccination, patient experienced Granuloma Annulare, Scalp Necrosis, and Ischemic Optic Neuropathy, but Optic Neuropathy is not the main disease. |
|  | Hassman LM, DiLoreto DA. Immunologic factors may play a role in herpes simplex virus 1 reactivation in the brain and retina after influenza vaccination. | After influenza vaccination, patient experienced both brain and retina syndrome, but ocular disease is not the main disease. |
|  | Blanco-Marchite CI, Buznego-Suárez L, Fagúndez-Vargas MA, Méndez-Llatas M, Pozo-Martos P. Miller fisher syndrome, internal and external ophthalmoplegia after flu vaccination. | After influenza vaccination, the patient experienced Miller fisher syndrome, a neurological disease, whose main clinical manifestations are neuromuscular. |
|  | Shoamanesh A, Chapman K, Traboulsee A. Postvaccination Miller Fisher syndrome. | After influenza vaccination, the patient experienced Miller fisher syndrome, a neurological disease, whose main clinical manifestations are neuromuscular. |
|  | Fredette MJ, De Serres G, Malenfant M. Ophthalmological and Biological Features of the Oculorespiratory Syndrome after Influenza Vaccination. Clinical Infectious Diseases 2003; 37(8): 1136-8 | After influenza vaccination, the patient experienced Oculorespiratory Syndrome |
| Implausible/ insufficient individual data | Geier MR, Geier DA. A case-series of adverse events, positive re-challenge of symptoms, and events in identical twins following hepatitis B vaccination: Analysis of the Vaccine Adverse Event Reporting System (VAERS) database and literature review. Clinical and experimental rheumatology 2004; 22(6): 749-55. | Case series lacking personal information |
|  | Fraunfelder FW, Suhler EB, Fraunfelder FT. Hepatitis B vaccine and uveitis: An emerging hypothesis suggested by review of 32 case reports. Cutaneous and ocular toxicology 2010; 29(1): 26-9. | Case series lacking personal information |
|  | Holt HD, Hinkle DM, Falk NS, Fraunfelder FT, Fraunfelder FW. Human papilloma virus vaccine associated uveitis. Current drug safety 2014; 9(1): 65-8. | Case series lacking personal information |
|  | Grillo AP, Fraunfelder FW. Keratitis in association with herpes zoster and varicella vaccines. Drugs of Today 2017; 53(7): 393-7 | Case series lacking personal information |
|  | Lim SA. Two cases of postvaccination optic neuritis/multiple sclerosis; coincidence or causative? Multiple Sclerosis 2012; 18(4): 541. | Case series lacking personal information |

Supplementary Table 5: Summary of basic information of all 105 studies (122 patients), 2003-2023

| Reference | Vaccines | Diagnosis | Vaccine category | Trade names | Ethnicity/  Country of publication | Age  /Gender | Important symptoms/signs/lab tests/medical history | Interval^ | Treatment | Outcome/Follow-up | Long-term vax |
| --- | --- | --- | --- | --- | --- | --- | --- | --- | --- | --- | --- |
| **Uveitis** | | | | | | | | | | | |
| *Uveitis anatomically classified* | | | | | | | | | | | |
| Marinho  et al  (2019)(1) | Yellow Fever vaccine | Asymmetric diffuse uveitis (Bilateral) | Live attenuated | NR | Brazilian/Caucasian | 50/F | fever, hypoxia, and nausea | 4 d | po. steroids | Complete recovery/ 40 d | - |
| Volkov  et al (2020)(2) | Yellow Fever/HAV/Meningitis vaccine | Viscerotropic disease followed by acute anterior and intermediate uveitis (Bilateral) | Live attenuated  (17D-204; Yellow fever) | VAQTA(HAV) | France/NR | 37/M | Fever, cough, dyspnea, malaise, sore throat, non-bloody diarrhea, and morbilliform skin rash of the chest/YFV RNA (+) | 2w | io. /gtt. Steroids | Recovering  /  NR | - |
| Biancardi et al (2019)(3) | Yellow Fever vaccine | Anterior uveitis (Right) | Live attenuated | NR | Brazil/NR | 35/F | None | 10 d | gtt. steroids | Complete recovery/NR | - |
|  |  | Intermediate uveitis (Left) | Live attenuated |  |  | 21/F | low fever, body ache, and mild headache | 14 d | po. steroids | Complete recovery/6 w | - |
| Richards et al (2021)(4) | VZV vaccine | Recurrent anterior and mild intermediate uveitis (Bilateral) | Recombinant | Shingrix | USA/NR | 69/M | Headache | 1 m | po. valacyclovir/gtt. steroids | Complete recovery/1 m | - |
|  |  | Recurrent anterior uveitis (Left) |  |  | USA/NR | 70/F | None | 2w | po. valacyclovir, po/gtt. steroids | Complete recovery/6 w | - |
|  |  | Recurrent multifocal choroiditis  (Bilateral) |  |  | USA/Caucasian | 57/F | Arm swelling at the injection site, chills, malaise, fever, and tinnitus/ immunosuppressant for multifocal choroiditis | 24 h | po. steroids and continued methotrexate | Stability/2 m | Long-term drug therapy |
| Sawai et al (2016)(5) | HPV vaccine | Anterior uveitis/ TINU Syndrome (Bilateral) | Recombinant | NR | Japan/NR | 14/F | Fever, general malaise, low back pain | 4 d | io. /gtt. /systemic steroid | Risk of long-term side effects/3 y | long-term steroids treatment for uveitis |
|  |  |  |  |  |  | 14/F | NR | 10 w | io. /gtt. steroid | Complete recovery/NR | - |
| Sham et al (2012)(6) | VZV vaccine | Exacerbation of anterior uveitis (right) | Live attenuated | Shingrix | USA/Caucasian | 86/M | Medical history of HZO with anterior uveitis | 3 w | po. Valacyclovir, gtt. steroids | Complete recovery/NR | - |
| Khalifa et al (2010)(7) | HPV vaccine | Ampiginous choroiditis (Bilateral) | Quadrivalent/ recombinant | NR | USA/NR | 17/F | None | 3 w | po. steroids | Stability/3 m | - |
| Wells et al (2009)(8) | Influenza vaccine- | Panuveitis (Bilateral) | NR | NR | USA/NR | 70/M | NR | 1 d | io. /gtt. steroids | Complete recovery/3 m | - |
| Sedaghat et al (2007)(9) | MMR vaccine | Panuveitis (Bilateral) | Live attenuated | NR | Iran/NR | 17/F | Fever, chills, skin rash and knee arthritis | 5 d | po. /gtt. steroids | Complete recovery/6 m | - |
| Chen, Y.-H. et al (2014)(10) | HPV vaccine | Panuveitis (Bilateral) | Quadrivalent/ recombinant | Gardasil | Taiwan/Asian | 27/F | Bilateral knee pain with morning stiffness, erythematous papules on the bilateral anterior legs, vertigo, and hearing impairment | 4 d | io. / po. steroids | Complete recovery/2 y | - |
| Rothova et al (2011)(11) | Influenza vaccine- | Panuveitis  (Bilateral) | Live attenuated | NR | Netherlands/  NR | 60/M | Not reported | 4 d | systemic/io. steroids | Recovering/NR |  |
| Heydari-Kamjani et al (2019)(12) | VZV vaccine | Uveitis sarcoidosis  (Bilateral) | Recombinant | Shingrix | USA/Caucasian | 53/F | Headaches | 4 d | Gtt. steroids | Complete recovery/NR | - |
| *Diseases involving the uvea and other ocular structure* | | | | | | | | | | | |
| Dansingani et al (2015)(13) | HPV vaccine | Panuveitis and ERD (Bilateral) | Quadrivalent/ recombinant | Gardasil | USA/Caucasian | 20/F | HLA-DRB1*0405(+) | 3 w | po. steroids | Complete recovery/5 m | - |
| Ye, H. et al (2020)(14) | HPV vaccine | Posterior uveitis resembling Harada disease (Bilateral) | Divalent/recombinant | Cervarix | China/Asian | 29/F | HLA-DR04&07(+) | 7 d after the third dose | io. /po. steroids | Complete recovery/4 m | - |
| Kong et al (2022)(15) | HPV vaccine | Posterior Harada disease-like uveitis  (Bilateral) | Quadrivalent/ recombinant | Gardasil | China/Asian | 37/F | None | 10 dafter the third dose | gtt. steroids | Complete recovery/3 m | - |
| Naseri et al (2003)(16) | VZV vaccine | Anterior Uveitis  (Left) | Live attenuated | NR | USA/NR | 9/M | Rash in left face, wild-type VZV DNA (+) | 3 y | po acyclovir/gtt. steroids | Stability/NR | - |
| Lin et al (2009)(17) | VZV vaccine | Keratouveitis  (Left) | Live attenuated | NR | USA/NR | 16/M | left-sided headache, Hutchinson’s sign | 8 y | po. anti-viral drugs/po. steroids, cyclopentolate | complete recovery/2 m | - |
| Hwang et al (2013)(18) | VZV vaccine | Reactivation of HZ keratouveitis  (Right) | Live attenuated | zostavax | USA/NR | 63/M | NR | 2 w | po. acyclovir / io. steroids/ cyclopentolate | Risk of long-term side effects/5 y | long-term steroids treatment/ punctate epithelial keratopathy |
| Kazuki Kuniyoshi et al (2017)(19) | Influenza  Pneumococcal /MMR vaccine | Acute vision loss with ERD/chorioretinitis  (Bilateral) | NR | NR | Asian/Japan | 13 m/M | Fever, respiratory tract infection; MMR antibody（+） | 24 d | steroids pulse-no response-tacrolimus | Risk of long-term side effects/9 m | Not pursue objects |
| Tao et al (2011)(20) | Influenza vaccine- | Posterior Uveitis and ERD (right) | Live attenuated | NR | China/Asian | 10/M | None | 10 d | iv./po. steroids | Complete recovery/1 m | - |
|  |  | Posterior uveitis and ERD (Bilateral) | Live attenuated | NR | China/Asian | 47/F | High fever, bilateral headache | 2 d | iv./po. steroids | Risk of long-term side effects /NR | - |
| Williams et al (2015)(21) | Influenza vaccine- | Retinal artery vasculitis  (Right) | Live attenuated | Influvac | UK/Caucasian | 78/F | Right-sided headache | 8 w | Gtt. steroids | Complete Recovery/9 m | - |
| Manusow et al (2015)(22) | Influenza vaccine- | Panuveitis with OIS  (Bilateral) | Live attenuated | NR | Canada/Caucasian | 49/F | Polyarthritis, fever, tender cervical lymphadenopathy | 4 d | iv/po steroids | Risk of long-term side effects/1 y | No light perception (Left) |
|  |  | Panuveitis with OIS (right) | Live attenuated | NR | Canada/Caucasian | 57/M | Mild jaundice, confusion and disorientation to place and time/ESR-84(+) | 3 d | iv./po. steroids | Recovering/3 m | - |
| Ferrini et al (2013)(23) | MMR vaccine | Anterior uveitis (Left) with iris heterochromia and cataract | Live attenuated | Priorix | France/Caucasian | 12m/F | HLA-B51, rubella IgG (+). | 3 m | Systemic/gtt /io. steroids +cataract extractio.n | Completely recovery/3 m | - |
| Host et al (2017)(24) | VZV vaccine | ARN  (Bilateral) | live attenuated | Zostavax | Australia/NR | 76/M | Diabetes, B-cell chronic lymphocytic leukemia and adult vitelliform macular dystrophy/wild-type VZV DNA (+) | 1 w | local and systemic antiviral therapy and steroids--developed ARN--Vitrectomy with insertion of silicone oil | NR | - |
| Charkoudian et al (2011)(25) | VZV vaccine | ARN (Left) | Live attenuated | Varivax/  zostavax | USA/NR | 77/F | VZV DNA (+)/diabetes mellitus | 6 d | po/iv antiviral drugs, vitrectomy | NR/NR | - |
|  |  | ARN  (Bilateral) | Live attenuated | Varivax/  zostavax | USA/NR | 80/M | rash and fever/ VZV DNA (+), immunosuppressant use for renal transplantation | 2 m | po/iv antiviral drugs, io. foscarnet, bilateral vitrectomy | NR/NR | - |
| Gonzales et al (2012)(26) | VZV vaccine | ARN  (Bilateral) | Live attenuated | Varivax/  zostavax | USA/NR | 20/M | Oka strain VZV DNA (+)/immunosuppressant for an inflammatory gastroenteropathy | 1 m | io. foscarnet, antiviral drugs, pars plana vitrectomy | NR/NR | - |
|  |  |  |  |  |  |  |  |  |  |  |  |
| Menghini et al (2021)(27) | VZV vaccine | ARN with obliterative angiopathy  (Left) | Live attenuated | Varivax/  zostavax | Australia  /Caucasian | 76/M | Insulin-dependent diabetes mellitus, chronic lymphocytic leukemia/ wild-type VZV DNA (+) | 2 d | io. foscarnet, iv. /po. antiviral drugs, iv. /po. steroids | Risk of long-term side effects/few months | Left eye visual acuity dropped to perception only |
| Chen RI et al (2020)(28) | VZV vaccine | ARN  (Left) | Recombinant | Varivax/  zostavax | USA/NR | 65/F | Immunomodulator for multiple myeloma/wild-type VZV DNA (+) | 6 w | io. foscarnet, iv. /po. antiviral drugs | Complete recovery/  19 w | - |
| Ali et al (2021)(29) | VZV vaccine | ARN(Left) | Live attenuated | Zostavax | USA/ Caucasian | 63/M | VZV DNA (+) | 2 m | po. steroids | Risk of long-term side effects/NR | - |
| Heath et al (2017)(30) | VZV vaccine | ARN(Left) | Live attenuated | Zostavax | UK/  Caucasian | 78/F | Oka strain VZV DNA (+)/immunosuppressant for autoimmune diabetes | 6 w | po. valaciclovir, gtt. steroids, pars plana vitrectomy | Stability/2 w | - |
| Weinlander et al (2019)(31) | VZV vaccine | ARN(Left) | Live attenuated | Zostavax | USA/NR | 64/M | Wild-type VZV DNA (+)/metabolic syndrome and impaired glucose tolerance | 16 m | po. Valacyclovir, po. /gtt. steroids | Complete recovery/6 m | - |
|  |  | ARN(Left) | Live attenuated | Zostavax | USA/NR | 62/M | Wild-type VZV DNA (+)/Cirrhosis and diabetes mellitus type 2 | 7 m | Po. Valacyclovir, gtt. steroids | Recovering/6 m | - |
| Wong et al (2019)(32) | MMR vaccine | AIBSES (Right) | Live attenuated | NR | USA/NR | 29/F | None | 20 d | None | Stability/6 w | - |
| Quinones et al_(2020)(33) | Influenza vaccine | AIBSES (Right) | NR | Afluria | USA/Hispanic | 57/F | type-2 diabetes mellitus | 11 d | po. Steroids | Risk of long-term side effects/1 y | No significant improvement was noted on her visual field examination |
| Gonome et al (2016)(34) | Influenza vaccine | APMPEE (Bilateral) | NR | NR | Japan/NR | 30/F | Fever, cough, and nausea | 17 d | Initiate iv. /gtt. NSAIDs/gtt. steroids | Complete recovery/1 m | - |
| Branisteanu et al (2015)(35) | Influenza vaccine- | APMPEE (Bilateral) | NR | NR | Romania/NR | 18/F | Intermittent headaches | 14 d | po. steroids | Stability/5 y | - |
| Mendrinos et al (2010)(36) | Influenza vaccine- | APMPEE (Bilateral) | NR | NR | Switzerland /Caucasian | 27/M | Flu-like symptoms | 14 d | po. steroids | Stability/3 m | - |
| Fine et al (2010)(37) | VZV vaccine | APMPEE (Bilateral) | Live attenuated | Varivax | US/Caucasian | 11/F | Severe headaches and tinnitus/VZV Ab (+) | 10 d | po. steroids | Complete recovery/1 y | - |
| Kraemer et al (2022)(38) | Combined use of multiple vaccines | APMPPE（Left） | NR | NR | USA/NR | 25/F | HLA-B*40 and HLA-DB1*15 alleles（+） | 3 w | io. steroids | complete recovery/  several months | - |
| Ng et al_(2020)(39) | Influenza vaccine | MEWDS (Right) | Recombinant | Flucelvax | USA/Caucasian | 34/M | myopic-photorefractive keratectomy | 2 w | None | complete recovery/4 w | - |
| Ogino et al  (2014)(40) | HPV vaccine | MEWDS (Left) | Bivalent/ recombinant | Cervarix | Japan/Asian | 16/F | Throat pain, headache | 2 w after the second dose | iv. steroids and antihistamine | Risk of long-term side effects/2 y | FA revealed recurrent leakage |
| Goyal et al (2013)(41) | Influenza vaccine | MEWDS (Right) | NR | NR | USA/Caucasian | 53/M | Not reported | 10 d | None | Complete recovery/1 m | - |
| Abou-Samra et al (2019)(42) | Influenza vaccine | MEWDS  (Right) | NR | NR | USA/NR | 27/F | Fever, rash, oral ulcers, arthralgias, headache, or vertigo. | 14 d | None | Stability/8 w | - |
| Fine et al (2001)(43) | HAV | MEWDS  (Left) | Inactivated | NR | Brazilian /Caucasian | 30/M | Not reported | 13 d after booster vaccination | None | Complete recovery / 6 w | - |
| Yang et al (2008)(44) | Rabies vaccine | MEWDS  (Left) | Inactivated | Rabipur | China/Asian | 33/F | None | 7 d after the third dose | io. steroids | Complete recovery/3 y | - |
| Stangos et al (2006)(45) | Combined use of multiple vaccines | MEWDS  (Left) | Inactivated | NR | Switzerland/ Caucasian | 50/F | None | 1 w | None | Spontaneous recovery/6 w | - |
| Cohen et al (2009)(46) | Combined use of multiple vaccines | MEWDS  (Left) | Recombinant | NR | USA/NR | 17/F | HLA-B27(+) | 1 m | None | Spontaneous recovery/2 m | - |
| Escott et al (2013)(47) | Combined use of multiple vaccines | Acute Multifocal choroiditis  (Right) | Inactivated | NR | USA/NR | 33/M | fever, rash, oral ulcers, arthralgias, headache, vertigo | 3 w | None | Stability/ 8 w | - |
| Sood et al (2019)(48) | HBV vaccine | VKH (Bilateral) | Recombinant | NR | USA/Caucasian | 43/M | Hearing loss, tinnitus. | 3 d | po. /io. steroids | Risk of long-term side effects/5 m | VKH with long-term steroids treatment |
| Murtaza et al (2022)(49) | Influenza vaccine | VKH (Bilateral) | Quadrivalent/ recombinant | NR | Filipino/Asian | 30/M | Headache, tinnitus, and HLA-DR4(+) | 2 d | io. /po. steroids | Stability/6 m | - |
| Kim et al (2016)(50) | Influenza vaccine | VKH (Bilateral) | Live attenuated | NR | Korea/Asian | 52/F | Tinnitus | 1 m | iv./po. steroid | Complete recovery/NR | - |
| Gallagher et al (2009)(51) | Influenza vaccine | VKH (Bilateral) | NR | NR | USA/NR | 44/F | Tinnitus | 1 m | iv./po. steroids with steroids-sparing and long-term immune-modulation | Complete recovery/NR | - |
| Campos et al (2021)(52) | Yellow Fever vaccine | VKH (Bilateral) | Live attenuated | NR | Brazil/  NR | 34/M | tinnitus, headache | 12 d after a booster dose | iv./po. steroids | Complete recovery/ 2 years | - |
| Pereima et al (2022)(53) | Yellow Fever vaccine | VKH (Bilateral) | Live attenuated | NR | NR | 45/M | tinnitus, headache | 2 w | iv./po. steroids | Stability/30 m | - |
| **Optic neuropathy** | | | | | | | | | | | |
| Papke et al (2017)(54) | Influenza vaccine | Optic neuropathy (Right) with optic disc edema | Trivalent /recombinant | Flucelvax | USA/NR | 55/F | bilateral knee effusions, muscle spasms and a facial rash, tinnitus | 2 d | iv. steroids | Risk of long-term side effects/1 y | OD vision remained at hand motions, with full depression of the right visual field Diffuse optic disc pallor. |
| Gemma Manasseh et al (2014)(55) | Influenza vaccine | Optic Neuropathy (Bilateral) | NR | NR | London/NR | 68/M | Type 2 diabetes mellitus, previous transurethral resection | 6 d | iv. steroids | Risk of long-term side effects/ 3 m | Inferior altitudinal visual field defect persisted |
| Manzotti et al (2010)(56) | MMR vaccine | Optic Neuropathy (Left) | Live attenuated | NR | Italian/Caucasian | 20months/M | Fever and cutaneous rash | 3 w | None | Complete recovery/20 d | - |
| Leiderman et al (2009)(57) | Influenza vaccine | Recurrent isolated sixth nerve palsy(Bilateral） | Inactivated | NR | USA/NR | 17 months/F | Not special | 3 w | Part-time occlusion therapy | Recovering/7 m | - |
| Grewal_Zeid et al (2014)(58) | HBV vaccine | Isolated abducens nerve palsy (Bilateral) | Recombinant | ENGERIX-B | Chicago/NR | 8 days/M | Not special | 4 d | Part-time occlusion therapy | complete recovery/2 m | - |
| Chon et al (2021)(59) | VZV Vaccine | Unilateral Ptosis, Proptosis, and Orbital Myositis（left） | Recombinant | NR | USA/Caucasian | 58/F | Fever, chills, diaphoresis, headache, and prickly sensation | 3 d | iv antibiotics, iv steroids | completed recovery/15 d | - |
| Kim et al_(2021)(60) | Influenza vaccine | Oculomotor Palsy (Left) | Inactivated | NR | Korea/Asian | 25/F | Not special | 2 w | iv. Steroids | complete recovery/4 w | - |
| Alfred Basilious et al (2020)(61) | H1N1 Influenza Vaccine | Ptosis (Bilateral) | Quadrivalent/  recombinant | Flulaval Tetra | Canada/Caucasian | 59/M | None | 2 h | None | complete recovery/1 m | - |
| Belliveau et al_(2011)(62) | H1N1 influenza vaccine | orbital inflammatory syndrome(left) | adjuvanted H1N1 pandemic influenza vaccine | Arepanrix | Canada/Caucasian | 45/M | Not special | Few hours | po. /io. steroids | complete recovery/1 y | - |
| Tan, Funda Uysal et al (2010)(63) | Influenza vaccine | Optic neuritis  (Bilateral) | Trivalent/  recombinant | NR | Turkey/NR | 55/F | Anticoagulant therapy because of atrial fibrillation. | 3 w | iv./po.steroids | complete recovery/6 m | - |
| Engels et al (2023)(64) | Monkeypox virus | Optic neuritis (Bilateral) | DNA vaccination | NR | German/NR | 52/M | MOG-IgG (+) | 10 d | Steroids | complete recovery/  22 w | - |
| Seigo Korematsu et al (2014)(65) | Influenza vaccine | Retrobulbar pain (Bilateral) | Trivalent/  recombinant | NR | Japan/Asian | 11 years and 6 months/F | Allergic disease, Elevated serum anti-phosphatidylcholine IgG antibodies | 2 d | Steroids pulse | Risk of long-term side effects/NR | - |
|  |  | Optic neuritis (Bilateral) |  | NR | Japan/Asian | 12 years and 9 months/F | myelin basic protein elevated, oligoclonal band IgG (+) | 1 d after second dose |  | Recovering/  NR | - |
| O'Dowd et al (2015)(66) | Combined use of multiple vaccines | Optic neuritis (Bilateral) | Inactivated | NR | Ireland/NR | 51/M | Not special | 2 w | iv./po. steroids /antibiotics  /Plasma exchange/immunoglobulin | Risk of long-term side effects/2 y | Bilateral blindness |
| Jun Fraunfelder et al (2018)(67) | Influenza vaccine | Asymmetric optic neuritis (Bilateral) | Inactivated | NR | Indian/Caucasian | 23/F | headache | 2 w | iv./po. steroids /plasma exchange | Recovering/  NR | - |
| Crawford et al (2013)(68) | Influenza vaccine | Optic Neuritis (Bilateral) | Trivalent/  recombinant | NR | Russia/NR | 13/M | Asthma and childhood obesity | 2 w | iv. Steroids | Complete recovery/3 months | - |
| Joshi et al (2016)(69) | MMR  vaccine | Optic Neuritis (Bilateral) | Live attenuated (Edmonston Zagreb strain) | NR | India/NR | 8/M | fever and headache | 21.5 h | io. /iv. / po. steroids/antibiotics | Complete recovery/5 m | - |
| DiMario et al (2010)(70) | HPV vaccine | Chiasmal Neuritis (Bilateral) | Recombinant | NR | NR | 16/F | Headache | 10 d | iv. Steroids, plasma exchanges | Risk of long-term side effects/18 mo | Light and movement from the left eye only |
| Arshi et al (2004)(71) | MMR vaccine | Optic Neuritis (Bilateral) | NR | NR | Iran/Caucasian | 16/M | vaso-vagal shock | few hours | po. /iv. steroids | Stability/NR | - |
| Saxena et al (2005)(72) | Rabies vaccine | Optic Neuritis (Bilateral) | Inactivated | Rabipur | India/Caucasian | 56/M | headache | 1 d after the third dose | iv. steroids | complete recovery/4 m | - |
| Agarwal et al (2020)(73) | Rabies vaccine | Optic Neuritis (Bilateral) | inactivated | RabAvert | India/Caucasian | 15/M | None | 3 d after the Fourth dose | iv steroids | Complete recovery/NR | - |
| Wang et al (2021)(74) | Rabies vaccine | Optic Neuritis (Bilateral) | Inactivated | NR | Asian | 23/M | Headache | 1 d after the fourth dose | iv./po. steroids | completed recovery/3 m | - |
| Erguven et al (2009)(75) | HBV vaccine | Optic Neuritis (left eye) | Recombinant | NR | turkey/Caucasian | 9/F | None | 1 w | pulsed-dose steroids | Complete recovery/NR | - |
| Sudarshan et al (2012)(76) | Hepatitis A vaccine | Retrobulbar optic neuritis(left) | Live attenuated | Not reported | Asian | 39/M | HIV infection | 12 d | iv./po. steroids | stability/3 y | - |
| Moradian et al (2008)(77) | MMR  Vaccine | Optic neuritis(right) | recombinant vaccine | Attenuvax; Meruvax | Caucasian/Iran | 17/M | Not special | 7 h | None | Complete recovery/3 w | - |
|  |  | Optic Neuritis (Bilateral) | recombinant vaccine | Attenuvax; Meruvax | Caucasian/Iran | 15/M | Headache and dizziness, a mild vasovagal shock | 6 h | iv. Steroids | Recovering/  NR | - |
| Gupta et al (2004)(78) | Rabies vaccine | Optic Neuritis (Bilateral) | inactivated vaccine | NR | Indian/ Caucasian | 15/M | Headache | 11 d | po. Steroids | Complete recovery/NR | - |
| Han et al (2014)(79) | VZV vaccine | Optic Neuritis (Bilateral) | Live attenuated | Zostavax | Korea/Asian | 55/F | ANA (+) | 2 w | iv. /po. steroids | Complete recovery/NR | - |
|  | VZV vaccine | Optic neuritis(right) | Live attenuated | Zostavax | Korea/Asian | 44/F | Not special | 1 w | iv. /po. steroids | Recovering/NR | - |
| Rubinov et al (2012)(80) | Influenza vaccine | Optic neuritis（Bilateral） | Quadrivalent/  recombinant | Vaxigrip Tetra | Istrael/ Caucasian | 18/M | Headache | 2 w | iv./po. steroids | Complete recovery/3 m | - |
| Dadeya et al (2004)(81) | Rabies vaccine | Retrobulbar neuritis（left） | Inactivated | Semple vaccine | India/ Caucasian | 31/M | Ascending myelitis. | 9 d | iv./po. steroids | complete recovery/8 w | - |
| Lapphra et al (2011)(82) | Influenza  Vaccine | Bilateral optic neuritis | Monovalent/ recombinant | Arepanrix | Filipino/Asian | 2/M | Mycoplasma IgM (+) | 6 d after second dose | iv. steroids | Complete recovery/6 m | - |
| chang et al (2016)(83) | HPV vaccine | NMOSD (Right) | Inactivated | Gardasil | Korea/Asian | 30/F | NMO-IgG (+) | 3 d after second recurrence | iv. steroids | Risk of long-term side effects/12 m | persistent disturbance of visual acuity. |
| Esmanhotto et al_(2021)(84) | Yellow Fever vaccine | NMOSD (Left) | Live attenuated | NR | Brazil/NR | 26/M | Demyelinating disease | 2 w | iv./po. Steroids | complete recovery/1 y | - |
| Menge et al (2012)(85) | HPV vaccine | NMO(Left) | Quadrivalent/  recombinant | Gardasil | Germany/NR | 17/F | NMO-IgG (+)/neuromyelitis | 9 m after third dose | iv. steroids/iv. rituximab and plasma exchange | Risk of long-term side effects /NR | - |
|  |  | NMO(Left) |  |  |  | 14/F | NMO-IgG (+)/neuromyelitis | 5 m after third dose | iv. steroids/iv. rituximab | completed recovery/NR | - |
|  |  | NMO(Bilateral) |  |  |  | 13/F | spinal MRI lesions | NR | no response to iv. steroids-rituximab | NR/NR | - |
|  |  | NMO(Bilateral) |  |  |  | 18/F | back pain, leg weakness | 8m after second dose | NR | NR/NR | - |
| Cho et al_(2019)(86) | influenza vaccine | NMOSD(Left) | Quadrivalent/  recombinant | NR | Korea/Asian | 38/F | severe neck and back pain with urinary retention | 3 d | iv./po. steroids | completed recovery  /12 m | - |
| Heekin et al (2015)(87) | HBV+Tdap | NMOSD  (Bilateral) | Hepatitis B vaccination(recombinant)+Tdap | NR | America  /Caucasian | 28/M | Neuromyelitis with fatigue, loss of balance | 11 d | iv steroids and plasma exchange | Stability/9 m | - |
| Younes et al (2021)(88) | VZV vaccine | Optic neuritis （Bilateral） | Subunit vaccination | Shingrix | USA/Caucasian | 51/F | Hypertension | 11 d | iv./po. steroids | complete recovery/12 m | - |
| Otiv et al (2022)(89) | MMR  vaccine | Optic neuritis with ADEM(Bilateral) | Live attenuated | NR | India/NR | 11/F | High-grade fever, headache, drowsiness, irrelevant talk, urine retention, and generalized seizure | 2 d | iv./po. steroids, IV immunoglobulin, antibiotics and antiviral drugs | Complete recovery/3 y | - |
| **Retinal disease** | | | | | | | | | | | |
| Landa et al (2006)(90) | smallpox vaccine | Multiple branch retinal arteriolar occlusions and encephalopathy（Left） | Live attenuated | NR | Japan/NR | 53/M | Phonic hearing defect, arterial hypertension, and an old branch retinal vein occlusion (BRVO) | 10 d | iv. Steroids/immunoglobulins | Recovering  /3 m | - |
| Shah et al (2018)(91) | Influenza vaccine | Acute Macular Neuroretinopathy (Right) | NR | NR | USA/Caucasian | 42/F | Prolonged viral-like illness | 1 w | None | Recovering  /1 m | - |
| Liu et al_(2018)(92) | Influenza vaccine | Acute macular neuroretinopathy (right) | inactivated | Fluvirin | USA/Caucasian | 47/F | Raynaud's phenomenon, Barrett's esophagus and herpes labialis. | 9 d | po. valacyclovir  /Vitamin D supplements  /Omeprazole | Recovering  /3 w | - |
| Juncal et al_(2022)(93) | HBV vaccine | PAMM (Right) | Recombinant | NR | Canada/NR | 12/F | Not special | 4 w | None | Complete recovery  / 9 m | - |
| Kwok et al (2013)(94) | influenza vaccine | Retinal vasculitis (Right) | Inactivated | Pandemrix | UK/NR | 63/M | None | 4 w | io. Steroids | Complete recover  /1 m | - |
|  | DTP+  hepatitis A and typhoid | retinal vasculitis (Right) | Inactivated (HAV) | Revaxis（DTP）+Viatim（typhoid+HAV） | UK/NR | 63/M | None | 2 m | io. / po. Steroids | Recovering  /1 m | - |
| Pereima et al (2022)(53) | Yellow Fever vaccine | Diffuse retinal vasculitis (Bilateral) | Live attenuated | NR | NR | 10/M | Toxocaracanis IgG (+) | 2 w | iv./po. steroids, po methotrexate | Risk of long-term side effects/ 2 y | persistent subtle perivascular leakage |
|  | Yellow Fever vaccine | Diffuse retinal vasculitis (Bilateral) | Live attenuated | NR | NR | 9/F | None | 2 w | io. steroids, methotrexate, azathioprine, and cyclosporine | Risk of long-term side effects/ 2 y | Uveitis was still active and diffuse retinal vasculitis |
| Moysidis et al (2017)(95) | Yellow Fever/  meningitidis | Retinal vessel vasculopathy  (Right) | Live attenuated  (Yellow Fever) | NR | USA/Caucasian | 41/M | Diffuse, generalized headaches 2 months | 4 d | None | Recovering  /1 m | Retina atrophy |
| **corneal disease** | | | | | | | | | | | |
| Jabbour et al (2021)(96) | VZV  vaccine | herpes zoster ophthalmicus (HZO) reactivation (Left) | Recombinant | Shingrix | USA/NR | 78/F | Medical history of HZO | 1 w after second dose | po. valacyclovir | Stability/2 m | - |
| Nguyen et al_(2020)(97) | Influenza vaccine | Keratitis (Left) | Quadrivalent/ recombinant | Fluzone | USA/NR | 64/F | Two-week history of reactivation of herpes zoster dermatitis in left flank. | 2 w and 4 d | po. Valacyclovir, io. voriconazole. | Recovering  /3 m | - |
| Lu, T.J._et al (2022)(98) | VZV  vaccine | HZO keratitis reactivation (Right) | Inactivated | NR | USA/NR | 75/F | NR | 2.5 w | po. Valacyclovir, io. steroids | complete recovery/6 m | - |
| Jastrzebski et al (2017)(99) | VZV  vaccine | Recurrent corneal perforaton (Right) | live attenuated | Zostavax | Canada/NR | 67/F | varicella zoster virus（+） | 2 w | io. moxifloxacin, steroids, antiglaucoma/po  anti-viral | Recovering  /6 y | - |
| Lehmann et al _(2018)(100) | VZV  vaccine | Recurrent stromal keratitis (Left) | Inactivated | NR | USA/NR | 89/M | NR | 3 w | io. steroids | Recovering/NR | - |
| Khalifa et al (2010)(101) | VZV  vaccine | Recurrence of interstitial Keratitis (Left) | Live attenuated | Zostavax | California/NR | 50/F | NR | 35 d | po. Valacyclovir hydrochloride; io. Loteprednol | Stability/18 months | - |
| Krall_  Kubal et al (2014)(102) | VZV  vaccine | Reactivation of keratitis (Left) | live attenuated | Varivax | NR/Florida | 6/F | vesicular rash and left eyelid swelling. | 1 y after booster dose | io. steroids/  po. antiviral drug | Risk of long-term side effects /1 y | Long-term steroids treatment, with only mild subepithelial haze |
| **conjunctival disease** | | | | | | | | | | | |
| Jenkins et al (2004)(103) | OPV | Conjunctives  (Bilateral) | inactivated | NR | New Zealand/NR | 10 m/F | Fever, running nose, cough, vomiting/eye swab poliovirus（+） | 5 m after the third dose | NR | NR | - |
| Kitaichi _et al (2006)(104) | MMR  vaccine | Conjunctivitis  (Left) | Live attenuated | NR | Japan/NR | 43/M | Left neck lymphadenopathy,  redness of injected skin | 12 d | NR | NR | - |
| Nazir et al_  (2023)(105) | MMR Vaccine | Vasculitis and Associated Conjunctivitis | live attenuated | NR | USA/NR | 78/M | multiple myeloma | 2 w | NR | NR | - |

ˆ Time between last vaccination and initial ocular symptom/sign. HBV = hepatitis B virus; APMPPE = acute posterior multifocal placoid pigment epitheliopathy; MEWDS = multiple evanescent white dot syndrome; VKH = Vogt–Koyanagi–Harada; HPV = human papillomavirus; TINU = tubulointerstitial nephritis and uveitis; MMR = measles–mumps–rubella; VZV = varicella zoster virus; HAV = hepatitis A virus; OIS = orbital inflammatory syndrome; OS = left eye; OD = right eye; ERD = exudative retinal detachment; ESR = erythrocyte sedimentation rate; ARN: acute retinal necrosis; NSAIDs = non-steroid anti-inflammatory drugs; RPE = retinal pigment epithelium; HLA = human leukocyte antigen; io/ir= intra-orbital/retrobulbar; iv = intravenous; gtt = eyedrops; po = orally; NR = not reported; Ab = antibody; HZO = herpes zoster ophthalmic; FA = fluorescein angiography; NMOSD=neuromyelitis optica spectrum disorder; NMO=neuromyelitis optica; ADEM=Acute Disseminated Encephalo-Myelitis; PAMM=paracentral acute middle maculopathy; DTP=diphtheria, tetanus and polio; OPV=oral poliovirus type 2 vaccine

Supplementary Table 6: Vaccines Potentially Implicated in Various Ocular Complications

| Ocular diseases | Trade names | Generic vaccine |
| --- | --- | --- |
| Uveitis anatomically classified | 17D-204(Yellow Fever); Shingrix (Varicella);  Zostavax (Zoster); Gardasil (HPV); | Yellow Fever (Live attenuated)  Zoster (Recombinant); HPV(Recombinant)  Zoster (Live attenuated)  Influenza H1N1(Live attenuated) |
| Diseases involving the uvea and other ocular structure | Gardasil (HPV); Cervarix(HPV); Zostavax (Zoster); Afluria,Fluvax (Influenza); Priorix (MMR); Shingrix (Varicella); Rabipur (Rabies);  Varivax(Varicella); | HPV(Recombinant); Zoster (Live attenuated)  Varicella (Live attenuated)  Measles-Rubella vaccination (Live attenuated)  Influenza H1N1(Live attenuated); Zoster (Recombinant); HBV(Recombinant)  Influenza (Recombinant); HAV (inactivated)  Rabies(inactivated); Moneypox virus (DNA); |
| Optic neuropathy | Flucelvax,Aventis-Pasteur,Phuket (Influenza); ENGERIX-B(HBV); Shingrix (Varicella); Rabipur (Rabies); Attenuvax (Measles); Meruvax(rubella); Zostavax (Zoster); Vaxigrip(Influenza); Gardasil (HPV); | Influenza (Recombinant); Measles-Rubella vaccination (Live attenuated); HBV(Recombinant); Monkeypox (DNA); HAV (inactivated); HPV(Recombinant); Rabies(inactivated);  Zoster (Live attenuated); Influenza H1N1(Recombinant); Yellow Fever (Live attenuated) |
| Retinal disease | Pandemrix,Fluvirin (Influenza), VAQTA(HAV) | Yellow Fever (Live attenuated)  Smallpox (Live attenuated)  HBV(Recombinant); HAV (inactivated); Influenza, swine influenza (inactivated) |
| corneal disease | Fluzone(Influenza); Zostavax (Zoster); Varivax(Varicella); Shingrix (Zoster) | Zoster (Recombinant); Influenza (Recombinant); Zoster (Live attenuated); Varicella (Live attenuated); |
| conjunctival disease | Biken, Tanabe(rubella) | oral poliovirus type 2 vaccine (inactivated)  Measles-Rubella vaccination (Live attenuated); |

Supplementary Table 7: Characteristics of long-term side effects and variables (88 patients, 128 eyes)

| Variable | N (%)/M±SD | |
| --- | --- | --- |
|  | Long-vax | Short-vax |
| **Age** | 35.20±24.28 | 35.43±21.48 |
| **Sex** |  |  |
| Male | 13 (50.0) | 50 (49.0) |
| Female | 13 (50.0) | 52 (51.0) |
| **Vaccine Generic** |  |  |
| Influenza virus vaccine | 5 (19.2) | 36 (35.3) |
| Varicella zoster virus (VZV) vaccine | 5 (19.2) | 16 (15.7) |
| Human papillomavirus (HPV) vaccine | 5 (19.2) | 11 (10.8) |
| Combined use of multiple vaccines | 5 (19.2) | 7 (6.9) |
| Yellow Fever virus vaccine | 4 (15.4) | 8 (7.8) |
| Hepatitis B virus (HBV) vaccine | 2 (7.7) | 3 (2.9) |
| #Others | 0 (0.0) | 21 (20.6) |
| **Diagnosis** |  |  |
| Uveitis | 13 (50.0) | 53 (52.0) |
| Optic neuropathy | 7 (26.9) | 38 (37.3) |
| Retinal disease | 5 (19.2) | 6 (5.9) |
| Corneal disease | 1 (3.8) | 5 (4.9) |
| **Affected eyes** |  |  |
| Left | 13 (50.0) | 54 (52.9) |
| Right | 13 (50.0) | 48 (47.1) |
| **Intervals** |  |  |
| ≤7 days | 13 (50.0) | 36 (35.3) |
| 7–30 days | 12 (46.2) | 58 (56.9) |
| >30 days | 1 (3.8) | 8 (7.8) |
| **Systemic symptoms** |  |  |
| With | 18 (69.2) | 60 (58.8) |
| Without | 8 (30.8) | 42 (41.2) |
| **Systemic steroids** |  |  |
| With | 25 (96.2) | 71 (69.6) |
| Without | 1 (3.8) | 31 (30.4) |

*Note. Baseline characteristics of the final cohort (88 patients, 128 eyes) after excluding two cases with delayed onset of ocular symptoms (>3 years after vaccination).*

*#Others include Measles-Mumps-Rubella (MMR) vaccine, Rabies vaccine, Hepatitis A virus (HAV) vaccine, Monkeypox virus, Smallpox virus, and Poliovirus vaccine.*

**Significant heterogeneity (P<0.05)*

**Supplementary Table 8. Factors associated with Long-vax among 128 eyes.**

| Variable | Univariate Analysis | | |
| --- | --- | --- | --- |
|  | B | 95% CI | p Value |
| **Sex (Female)** | 0.031 | -1.011-1.074 | 0.953 |
| **Vaccine Generic** | | | |
| Influenza virus vaccine | -0.717 | -1.944-0.510 | 0.252 |
| Varicella zoster virus (VZV) vaccine | 0.315 | -0.950-1.581 | 0.625 |
| Human papillomavirus (HPV) vaccine | 1.127 | -0.259-2.514 | 0.111 |
| Combined use of multiple vaccines | 0.861 | -0.636-2.357 | 0.260 |
| Yellow Fever virus vaccine | 0.579 | -1.151-2.308 | 0.512 |
| Hepatitis B virus (HBV) vaccine | 0.782 | -1.677-3.241 | 0.533 |
| #Others | - | - | - |
| **Diagnosis** | | | |
| Uveitis | -0.057 | -1.096-0.982 | 0.914 |
| Optic neuropathy | -0.324 | -1.476-0.828 | 0.582 |
| Retinal disease | 0.870 | -0.627-2.366 | 0.255 |
| Corneal disease | -0.194 | -2.407-2.018 | 0.863 |
| **Affected eyes (Left)** | 0.276 | -0.150-0.702 | 0.204 |
| **Intervals** | | | |
| ≤7 days[ref] |  |  |  |
| 7–30 days | -0.713 | -1.799-0.374 | 0.198 |
| >30 days | -1.106 | -3.324-1.112 | 0.328 |
| **Systemic steroids** | 2.192 | 0.117-4.267 | 0.038* |

*Note. Baseline characteristics of the final cohort (88 patients, 128 eyes) after excluding two cases with delayed onset of ocular symptoms (>3 years after vaccination).* *GEE logistic regression coefficients are shown (reference =short-vax).*

*#Others include Measles-Mumps-Rubella (MMR) vaccine, Rabies vaccine, Hepatitis A virus (HAV) vaccine, Monkeypox virus, Smallpox virus, and Poliovirus vaccine.*

**Significant heterogeneity (P<0.05)*

**Supplementary Table 9: Description of 26 eyes in various vaccinations and types of Long-vax**

| Type of Long-vax | *Disturbance of visual acuity | #Long-term drug therapy | ^Visual field defect | Persistent vascular leakage | Uveitis was still active with diffuse retinal vasculitis | Total number of eyes (n, %) |
| --- | --- | --- | --- | --- | --- | --- |
| Vaccine generic | | | | | | |
| HPV | 2 | 2 |  | 1 |  | 5 (19.2%) |
| Influenza | 2 |  | 3 |  |  | 5 (19.2%) |
| VZV | 1 | 4 |  |  |  | 5 (19.2%) |
| Combined use of multiple vaccines | 4 |  |  |  | 1 | 5 (19.2%) |
| Yellow Fever |  |  |  | 2 | 2 | 4 (15.4%) |
| HBV |  | 2 |  |  |  | 2 (7.7%) |
| Diagnosis | | | | | | |
| Uveitis |  |  |  |  |  | 13 (50.0%) |
| *Uveitis anatomically classified* |  | 2 |  |  |  | 2 |
| *Diseases involving the uvea and other ocular structure* |  |  |  |  |  | 11 |
| MFC |  | 2 |  |  |  | 2 |
| Posterior uveitis and ERD | 2 |  |  |  |  | 2 |
| VKH |  | 2 |  |  |  | 2 |
| AIBSES |  |  | 1 |  |  | 1 |
| ARN | 1 |  |  |  |  | 1 |
| Keratouveitsis |  | 1 |  |  |  | 1 |
| MEWDS |  |  |  | 1 |  | 1 |
| Panuveitis with OIS | 1 |  |  |  |  | 1 |
| Optic neuropathy |  |  |  |  |  | 7(26.9%) |
| Optic neuritis | 3 |  |  |  |  | 3 |
| *Sequential Non-Arteritic Anterior Ischemic Optic Neuropathy* |  |  | 2 |  |  | 2 |
| *Optic neuritis with myelitis or encephalitis* | 1 |  |  |  |  | 1 |
| *optic neuropathy with optic disc oedema* | 1 |  |  |  |  | 1 |
| Retinal disease |  |  |  |  |  | 5 (19.2%) |
| *Diffuse retinal vasculitis* |  |  |  | 2 | 2 |  |
| *Retinal vessel vasculopathy* |  |  |  |  | 1 |  |
| Corneal disease |  |  |  |  |  | 1 (3.8%) |
| *Reactivation of keratitis* |  | 1 |  |  |  |  |
| Total number of eyes (n, %) | 9(34.6%) | 8(30.8%) | 3(11.5%) | 3(11.5%) | 3(11.5%) | 26 (100.0%) |

*Abbreviation: HPV = human papillomavirus; VZV=varicella-zoster virus; HBV=Hepatitis B virus; MFC= multifocal choroiditis; ERD = exudative retinal detachment; VKH = Vogt–Koyanagi–Harada; AIBSES= acute idiopathic blind spot enlargement syndrome; ARN: acute retinal necrosis; MEWDS = multiple evanescent white dot syndrome; OIS = orbital inflammatory syndrome*

*Description: Two patients with bilateral involvement improved in one eye but not the other after treatment(22, 70)*

**1 No light perception (left);(22) 1 vision remained at hand motions (right),(54) 1 blindness (bilateral);(66) 1 Not pursue objects (Bilateral),(19) 1 dropped to perception only (left),(27) 1 persistent profound visual impairment, inconsistently identifying light and movement (left),(70)1 persistent disturbance of visual acuity (right).(83)*

*#Long-term drug therapy: 1 long-term steroid treatment for uveitis (bilateral),(5) 2 Long-term steroid treatments with punctate epithelial keratopathy and a mild subepithelial haze (unilateral),(18, 102) 1 long-term steroids treatment for Vogt–Koyanagi–Harada (bilateral);(48) 1 Intravitreal bevacizumab for a secondary choroidal neovascular membrane (bilateral);(4)*

*^No significant improvement on visual field examination (right),(33)* *inferior altitudinal visual field defect persisted (bilateral).*

**Reference**

1. Marinho PM, Nascimento H, Romano A, Muccioli C, Belfort R. Diffuse uveitis and chorioretinal changes after yellow fever vaccination: A re-emerging epidemic. *Int J Retina Vitreous*. 2019;5(1).10.1186/s40942-019-0180-0: 10.1186/s40942-019-0180-0

2. Volkov L, Grard G, Bollaert PE, Durand GA, Cravoisy A, Conrad M, et al. Viscerotropic disease and acute uveitis following yellow fever vaccination: A case report. BMC infectious diseases. 2020;20(1).10.1186/s12879-020-4838-x: 10.1186/s12879-020-4838-x

3. Biancardi AL, Moraes HV, Jr. Anterior and Intermediate Uveitis Following Yellow Fever Vaccination with Fractional Dose: Case Reports. Ocular immunology and inflammation. 2019;27(4):521-3.10.1080/09273948.2018.1510529: 10.1080/09273948.2018.1510529

4. Richards PJ, Wingelaar MJ, Armbrust KR, Kopplin LJ. Uveitis reactivation following recombinant zoster vaccination. American Journal of Ophthalmology Case Reports. 2021;23.10.1016/j.ajoc.2021.101115: 10.1016/j.ajoc.2021.101115

5. Sawai T, Shimizu M, Sakai T, Yachie A. Tubulointerstitial nephritis and uveitis syndrome associated with human papillomavirus vaccine. Journal of pediatric ophthalmology and strabismus. 2016;53(3):190-1.10.3928/01913913-20160405-04: 10.3928/01913913-20160405-04

6. Sham CW, Levinson RD. Uveitis exacerbation after varicella-zoster vaccination in an adult. Archives of Ophthalmology. 2012;130(6):793-4.10.1001/archophthalmol.2011.1881: 10.1001/archophthalmol.2011.1881

7. Khalifa YM, Monahan PM, Acharya NR. Ampiginous choroiditis following quadrivalent human papilloma virus vaccine. British Journal of Ophthalmology. 2010;94(1):137-9.10.1136/bjo.2009.159293: 10.1136/bjo.2009.159293

8. Wells GA, Shea B, O’Connell D, Peterson J, Welch V, Losos M, et al. The Newcastle-Ottawa Scale (NOS) for assessing the quality of nonrandomised studies in meta-analyses. 2000

9. Sedaghat M, Zarei-Ghanavati S, Shokoohi S, Ghasemi A. Panuveitis and dermal vasculitis following MMR vaccination. Eastern Mediterranean Health Journal. 2007;13(2):470-4

10. Chen YH, Chang YH, Lee YC. Panuveitis following administration of quadrivalent human papillomavirus vaccine. Tzu Chi Medical Journal. 2014;26(1):44-6.10.1016/j.tcmj.2012.06.003: 10.1016/j.tcmj.2012.06.003

11. Rothova A, de Groot JD, Mudrikova T. Reactivation of acute retinal necrosis after flu H1N1 vaccination. The British journal of ophthalmology. 2011;95(2):291.10.1136/bjo.2010.185983: 10.1136/bjo.2010.185983

12. Heydari-Kamjani M, Vante I, Uppal P, Demory Beckler M, Kesselman MM. Uveitis Sarcoidosis Presumably Initiated After Administration of Shingrix Vaccine. Cureus. 2019;11(6):e4920.10.7759/cureus.4920: 10.7759/cureus.4920

13. Dansingani KK, Suzuki M, Naysan J, Samson CM, Spaide RF, Fisher YL. Panuveitis with exudative retinal detachments after vaccination against human papilloma virus. Ophthalmic Surgery Lasers and Imaging Retina. 2015;46(9):967-70.10.3928/23258160-20151008-11: 10.3928/23258160-20151008-11

14. Ye H, Feng H, Zhao P, Fei P. Case Report: Posterior Uveitis after Divalent Human Papillomavirus Vaccination in an Asian Female. Optometry and vision science : official publication of the American Academy of Optometry. 2020;97(6):390-4.10.1097/OPX.0000000000001523: 10.1097/OPX.0000000000001523

15. Kong K, Ding X, Ni Y. Resolution of Harada disease-like uveitis after quadrivalent human papillomavirus vaccination: a case report. Human Vaccines and Immunotherapeutics. 2022;18(1):1-4.10.1080/21645515.2021.1953349: 10.1080/21645515.2021.1953349

16. Naseri A, Good WV, Cunningham ET, Jr. Herpes zoster virus sclerokeratitis and anterior uveitis in a child following varicella vaccination. American journal of ophthalmology. 2003;135(3):415-7.10.1016/s0002-9394(02)01957-8: 10.1016/s0002-9394(02)01957-8

17. Lin P, Yoon MK, Chiu CS. Herpes zoster keratouveitis and inflammatory ocular hypertension 8 years after varicella vaccination. Ocular immunology and inflammation. 2009;17(1):33-5.10.1080/09273940802491892: 10.1080/09273940802491892

18. Hwang CW, Steigleman WA, Saucedo-Sanchez E, Tuli SS. Reactivation of herpes zoster keratitis in an adult after varicella zoster vaccination. Cornea. 2013;32(4):508-9.10.1097/ICO.0b013e318277acae: 10.1097/ICO.0b013e318277acae

19. Kuniyoshi K, Hatsukawa Y, Kimura S, Fujino T, Ohguro H, Nakai R, et al. Acute Bilateral Photoreceptor Degeneration in an Infant After Vaccination Against Measles and Rubella. JAMA ophthalmology. 2017;135(5):478-82.10.1001/jamaophthalmol.2017.0380: 10.1001/jamaophthalmol.2017.0380

20. Tao Y, Chang LB, Zhao M, Li XX. Two cases of exudative retina detachment and uveitis following H1N1 influenza vaccination. Chinese medical journal. 2011;124(22):3838-40.10.3760/cma.j.issn.0366-6999.2011.22.044: 10.3760/cma.j.issn.0366-6999.2011.22.044

21. Williams GS, Evans S, Yeo D, Al-Bermani A. Retinal artery vasculitis secondary to administration of influenza vaccine. BMJ case reports. 2015;2015.10.1136/bcr-2015-211971: 10.1136/bcr-2015-211971

22. Manusow JS, Rai A, Yeh S, Mandelcorn ED. Two cases of panuveitis with orbital inflammatory syndrome after influenza vaccination. Canadian journal of ophthalmology Journal canadien d'ophtalmologie. 2015;50(5):e71-4.10.1016/j.jcjo.2015.05.016: 10.1016/j.jcjo.2015.05.016

23. Ferrini W, Aubert V, Balmer A, Munier FL, Abouzeid H. Anterior uveitis and cataract after rubella vaccination: A case report of a 12-month-old girl. Pediatrics. 2013;132(4):e1035-e8.10.1542/peds.2012-2930: 10.1542/peds.2012-2930

24. Host B, Chen F, Constable I, Tay-Kearney ML, Flexman J. Zostavax® vaccine triggering bilateral acute retinal necrosis due to wild-type varicella zoster virus. Clinical and Experimental Ophthalmology. 2017;45:147.10.1111/ceo.13054/full: 10.1111/ceo.13054/full

25. Charkoudian LD, Kaiser GM, Steinmetz RL, Srivastava SK. Acute retinal necrosis after herpes zoster vaccination. Archives of Ophthalmology. 2011;129(11):1495-7.10.1001/archophthalmol.2011.320: 10.1001/archophthalmol.2011.320

26. Gonzales JA, Levison AL, Stewart JM, Acharya NR, Margolis TP. Retinal necrosis following varicella-zoster vaccination. Archives of Ophthalmology. 2012;130(10):1355-6.10.1001/archophthalmol.2012.2255: 10.1001/archophthalmol.2012.2255

27. Menghini M, Raja V, Raiter J, Balaratnasingam C, Constable IJ. ACUTE RETINAL NECROSIS ASSOCIATED WITH HERPES ZOSTER VACCINATION. Retinal cases & brief reports. 2021;15(2):166-8.10.1097/icb.0000000000000761: 10.1097/icb.0000000000000761

28. Chen RI, Deaner JD, Srivastava SK, Lowder CY. Acute retinal necrosis following recombinant subunit varicella-zoster virus vaccine. American Journal of Ophthalmology Case Reports. 2020;20.10.1016/j.ajoc.2020.100962: 10.1016/j.ajoc.2020.100962

29. Ali A, Kirschenbaum MD, Sharma S, Wandel TL. ACUTE RETINAL NECROSIS AND CONTRALATERAL CUTANEOUS ERUPTION AFTER THE SHINGLES VACCINE. Retinal cases & brief reports. 2021;15(1):43-4.10.1097/icb.0000000000000729: 10.1097/icb.0000000000000729

30. Heath G, Depledge DP, Brown JR, Hale AD, Tutil H, Williams R, et al. Acute Retinal Necrosis Caused by the Zoster Vaccine Virus. Clinical Infectious Diseases. 2017;65(12):2122-5.10.1093/cid/cix683: 10.1093/cid/cix683

31. Weinlander EJ, Wang AL, Jaru-Ampornpan P, Altaweel MM, Nork TM. TWO CASES of ACUTE RETINAL NECROSIS DUE to VARICELLA ZOSTER DESPITE PRIOR SHINGLES VACCINATION. Retinal Cases and Brief Reports. 2019;13(3):241-3.10.1097/ICB.0000000000000567: 10.1097/ICB.0000000000000567

32. Wong M, Campos-Baniak MG, Colleaux K. Acute idiopathic blind spot enlargement syndrome following measles, mumps and rubella vaccination. Canadian Journal of Ophthalmology. 2019;54(4):e199-e203.10.1016/j.jcjo.2018.09.005: 10.1016/j.jcjo.2018.09.005

33. Quinones X, Ortiz J, Santos C, Oliver AL, Rodríguez J. Acute idiopathic blind spot enlargement syndrome following influenza vaccination. American Journal of Ophthalmology Case Reports. 2020;20.10.1016/j.ajoc.2020.100949: 10.1016/j.ajoc.2020.100949

34. Gonome T, Suzuki Y, Metoki T, Takahashi S, Nakazawa M. Acute posterior multifocal placoid pigment epitheliopathy and granulomatous uveitis following influenza vaccination. Am J Ophthalmol Case Rep. 2016;4:60-3.10.1016/j.ajoc.2016.08.008: 10.1016/j.ajoc.2016.08.008

35. Branisteanu D, Bilha A. ACUTE POSTERIOR MULTIFOCAL PLACOID PIGMENT EPITHELIOPATHY FOLLOWING INFLUENZA VACCINATION. Romanian journal of ophthalmology. 2015;59(1):52-8

36. Mendrinos E, Baglivo E. Acute posterior multifocal placoid pigment epitheliopathy following influenza vaccination. Eye. 2010;24(1):180-1.10.1038/eye.2009.68: 10.1038/eye.2009.68

37. Fine HF, Kim E, Flynn TE, Gomes NL, Chang S. Acute posterior multifocal placoid pigment epitheliopathy following varicella vaccination. British Journal of Ophthalmology. 2010;94(3):282-3+363.10.1136/bjo.2008.144501: 10.1136/bjo.2008.144501

38. Kraemer LS, Montgomery JR, Baker KM, Colyer MH. Acute posterior multifocal placoid pigment epitheliopathy after immunization with multiple vaccines. Retinal Cases and Brief Reports. 2022;16(1):16-9.10.1097/ICB.0000000000000959: 10.1097/ICB.0000000000000959

39. Ng CC, Jumper JM, Cunningham ET. Multiple evanescent white dot syndrome following influenza immunization - A multimodal imaging study. American Journal of Ophthalmology Case Reports. 2020;19.10.1016/j.ajoc.2020.100845: 10.1016/j.ajoc.2020.100845

40. Ogino K, Kishi S, Yoshimura N. Multiple evanescent white dot syndrome after human papillomavirus vaccination. Case Reports in Ophthalmology. 2014;5(1):38-43.10.1159/000358870: 10.1159/000358870

41. Goyal S, Nazarian SM, Thayi DR, Hammond F, Petrovic V. Multiple evanescent white dot syndrome following recent influenza vaccination. Canadian Journal of Ophthalmology. 2013;48(5):e115-e6.10.1016/j.jcjo.2013.03.002: 10.1016/j.jcjo.2013.03.002

42. Abou-Samra A, Tarabishy AB. Multiple Evanescent White Dot Syndrome Following Intradermal Influenza Vaccination. Ocular immunology and inflammation. 2019;27(4):528-30.10.1080/09273948.2017.1423334: 10.1080/09273948.2017.1423334

43. Fine L, Fine A, Cunningham ET, Jr. Multiple evanescent white dot syndrome following hepatitis a vaccination. Archives of ophthalmology (Chicago, Ill : 1960). 2001;119(12):1856-8.10.1001/archopht.119.12.1870: 10.1001/archopht.119.12.1870

44. Yang JS, Chen CL, Hu YZ, Zeng R. Multiple evanescent white dot syndrome following rabies vaccination: a case report. BMC ophthalmology. 2018;18(1):312.10.1186/s12886-018-0968-y: 10.1186/s12886-018-0968-y

45. Stangos A, Zaninetti M, Petropoulos I, Baglivo E, Pournaras C. Multiple evanescent white dot syndrome following simultaneous hepatitis-A and yellow fever vaccination. Ocular immunology and inflammation. 2006;14(5):301-4.10.1080/09273940600932311: 10.1080/09273940600932311

46. Cohen SM. Multiple Evanescent White Dot Syndrome After Vaccination for Human Papilloma Virus and Meningococcus. J Pediatr Ophthalmol Strabismus. 2009.10.3928/01913913-20090616-01: 10.3928/01913913-20090616-01

47. Escott S, Tarabishy AB, Davidorf FH. Multifocal choroiditis following simultaneous hepatitis a, typhoid, and yellow fever vaccination. Clinical Ophthalmology. 2013;7:363-5.10.2147/opth.s37443: 10.2147/opth.s37443

48. Sood AB, O'Keefe G, Bui D, Jain N. Vogt-Koyanagi-Harada Disease Associated with Hepatitis B Vaccination. Ocular immunology and inflammation. 2019;27(4):524-7.10.1080/09273948.2018.1483520: 10.1080/09273948.2018.1483520

49. Murtaza F, Pereira A, Mandelcorn MS, Kaplan AJ. Vogt-Koyanagi-Harada disease following influenza vaccination. American Journal of Ophthalmology Case Reports. 2022;26.10.1016/j.ajoc.2022.101516: 10.1016/j.ajoc.2022.101516

50. Kim M. Vogt-Koyanagi-Harada Syndrome following influenza vaccination. Indian journal of ophthalmology. 2016;64(1):98.10.4103/0301-4738.178141: 10.4103/0301-4738.178141

51. Gallagher MJ, Yilmaz T, Foster CS. Vogt-Koyanagi-Harada syndrome associated with bilateral serous macular detachments responsive to immunomodulatory therapy. Ophthalmic surgery, lasers & imaging : the official journal of the International Society for Imaging in the Eye. 2009;40(3):345-7.10.3928/15428877-20090430-25: 10.3928/15428877-20090430-25

52. Campos WR, Cenachi SPF, Soares MS, Gonçalves PF, Vasconcelos-Santos DV. Vogt–Koyanagi–Harada-like Disease following Yellow Fever Vaccination. Ocular immunology and inflammation. 2021;29(1):124-7.10.1080/09273948.2019.1661498: 10.1080/09273948.2019.1661498

53. Pereima RR, Bonatti R, Crotti F, Furtado JM, Lopes MH, Yamamoto JH, et al. Ocular Adverse Events following Yellow Fever Vaccination: A Case Series. Ocular immunology and inflammation. 2022;30(6):1425-9.10.1080/09273948.2021.1887279: 10.1080/09273948.2021.1887279

54. Papke D, McNussen PJ, Rasheed M, Tsipursky MS, Labriola LT. A Case of Unilateral Optic Neuropathy Following Influenza Vaccination. Seminars in ophthalmology. 2017;32(4):517-23.10.3109/08820538.2015.1120758: 10.3109/08820538.2015.1120758

55. Manasseh G, Donovan D, Shao EH, Taylor SR. Bilateral sequential non-arteritic anterior ischaemic optic neuropathy following repeat influenza vaccination. Case Reports in Ophthalmology. 2014;5(2):267-9.10.1159/000366472: 10.1159/000366472

56. Manzotti F, Menozzi C, Porta MR, Orsoni JG. Partial third nerve palsy after Measles Mumps Rubella vaccination. Italian journal of pediatrics. 2010;36(1):59.10.1186/1824-7288-36-59: 10.1186/1824-7288-36-59

57. Leiderman YI, Lessell S, Cestari DM. Recurrent isolated sixth nerve palsy after consecutive annual influenza vaccinations in a child. Journal of AAPOS : the official publication of the American Association for Pediatric Ophthalmology and Strabismus. 2009;13(3):317-8.10.1016/j.jaapos.2008.12.137: 10.1016/j.jaapos.2008.12.137

58. Grewal DS, Zeid JL. Isolated abducens nerve palsy following neonatal hepatitis B vaccination. Journal of AAPOS. 2014;18(1):75-6.10.1016/j.jaapos.2013.09.012: 10.1016/j.jaapos.2013.09.012

59. Chon BH, Hwang CJ, Perry JD. Unilateral Ptosis, Proptosis, and Orbital Myositis After Shingles Vaccination. Ophthalmic plastic and reconstructive surgery. 2021;37(3):E100-E3.10.1097/IOP.0000000000001847: 10.1097/IOP.0000000000001847

60. Kim BY, Yung Y, Kim GS, Park HR, Lee JJ, Song P, et al. Complete Oculomotor Palsy after Influenza Vaccination in a Young Healthy Adult: A Case Report. Case Reports in Neurology. 2021;13(1):35-9.10.1159/000511025: 10.1159/000511025

61. Basilious A, Jivraj I, Deangelis D. Acute Unilateral Ptosis and Myositis following the H1N1 Influenza Vaccine. Ophthalmic plastic and reconstructive surgery. 2020;36(1):e16-e7.10.1097/IOP.0000000000001517: 10.1097/IOP.0000000000001517

62. Belliveau MJ, Kratky V, Evans GA, Almeida DR, El-Defrawy S. Acute orbital inflammatory syndrome following H1N1 immunization. Canadian journal of ophthalmology Journal canadien d'ophtalmologie. 2011;46(6):552-3.10.1016/j.jcjo.2011.09.018: 10.1016/j.jcjo.2011.09.018

63. Tan FU, Akarsu C, Gullu R, Kansu T. Bilateral Optic Neuritis After Influenza Vaccination. Neuro-Ophthalmology. 2010;34(2):115-7.10.3109/01658101003725847: 10.3109/01658101003725847

64. Engels D, Mader S, Förderreuther S, Reindl M, Havla J, Meinl E, et al. MOG-IgG-Associated Bilateral Optic Neuritis in Temporal Relation to Monkeypox Vaccination. Annals of neurology. 2023;93(6):1216-7.10.1002/ana.26664: 10.1002/ana.26664

65. Korematsu S, Miyahara H, Kakita A, Izumi T. Elevated serum anti-phosphatidylcholine IgG antibodies in patients with influenza vaccination-associated optic neuritis. Vaccine. 2014;32(48):6345-8.10.1016/j.vaccine.2014.09.053: 10.1016/j.vaccine.2014.09.053

66. O'Dowd S, Bafiq R, Ryan A, Cullinane A, Costello D. Severe bilateral optic neuritis post hepatitis A virus (HAV) and typhoid fever vaccination. Journal of the neurological sciences. 2015;357(1-2):300-1.10.1016/j.jns.2015.06.061: 10.1016/j.jns.2015.06.061

67. Jun B, Fraunfelder FW. Atypical Optic Neuritis After Inactivated Influenza Vaccination. Neuro-Ophthalmology. 2018;42(2):105-8.10.1080/01658107.2017.1335333: 10.1080/01658107.2017.1335333

68. Crawford CM, Grazko MB, Raymond WRt, Rivers BA, Munson PD. Retrobulbar optic neuritis and live attenuated influenza vaccine. Journal of pediatric ophthalmology and strabismus. 2013;50(1):61.10.3928/01913913-20121108-01: 10.3928/01913913-20121108-01

69. Joshi J, Seth A, Aneja S, Singh AK, Aggarwal MK, Gupta N. Rapid onset optic neuritis following measles vaccine in India: Case report. Vaccine Reports. 2016;6:86-8.10.1016/j.vacrep.2017.01.001: 10.1016/j.vacrep.2017.01.001

70. DiMario FJ, Jr., Hajjar M, Ciesielski T. A 16-year-old girl with bilateral visual loss and left hemiparesis following an immunization against human papilloma virus. Journal of child neurology. 2010;25(3):321-7.10.1177/0883073809349322: 10.1177/0883073809349322

71. Arshi S, Sadeghi-Bazargani H, Ojaghi H, Savadi-Oskouei D, Hekmat S, Jastan M, et al. The first rapid onset optic neuritis after measles-rubella vaccination: case report. Vaccine. 2004;22(25-26):3240-2.10.1016/j.vaccine.2004.02.044: 10.1016/j.vaccine.2004.02.044

72. Saxena R, Sethi HS, Rai HK, Menon V. Bilateral neuro-retinitis following chick embryo cell anti-rabies vaccination - A case report. BMC ophthalmology. 2005;5.10.1186/1471-2415-5-20: 10.1186/1471-2415-5-20

73. Agarwal A, Garg D, Goyal V, Pandit AK, Srivastava AK, Srivastava MVP. Optic neuritis following anti-rabies vaccine. Tropical doctor. 2020;50(1):85-6.10.1177/0049475519872370: 10.1177/0049475519872370

74. Wang J, Tian M, Li F, Du X, Han Y, Ma P. Bilateral Optic Neuritis After Vero Cell-Derived Antirabies Vaccination. Journal of neuro-ophthalmology : the official journal of the North American Neuro-Ophthalmology Society. 2021;41(3):e300-e2.10.1097/wno.0000000000001077: 10.1097/wno.0000000000001077

75. Erguven M, Guven S, Akyuz U, Bilgiç O, Laloglu F. Optic Neuritis Following Hepatitis B Vaccination in a 9-year-old Girl. Journal of the Chinese Medical Association. 2009;72(11):594-7.10.1016/S1726-4901(09)70435-6: 10.1016/S1726-4901(09)70435-6

76. Sudarshan S, Huang EH, Lim PL, Leo YS, Lim SA. A case of postvaccination optic neuritis: coincidence or causative? Eye (London, England). 2012;26(11):1498.10.1038/eye.2012.146: 10.1038/eye.2012.146

77. Moradian S, Ahmadieh H. Early onset optic neuritis following measles-rubella vaccination. Journal of ophthalmic & vision research. 2008;3(2):118

78. Gupta V, Bandyopadhyay S, Bapuraj JR, Gupta A. Bilateral Optic Neuritis Complicating Rabies Vaccination. Retina (Philadelphia, Pa). 2004;24(1):179-81.10.1097/00006982-200402000-00033: 10.1097/00006982-200402000-00033

79. Han SB, Hwang JM, Kim JS, Yang HK. Optic neuritis following Varicella zoster vaccination: Report of two cases. Vaccine. 2014;32(39):4881-4.10.1016/j.vaccine.2014.07.023: 10.1016/j.vaccine.2014.07.023

80. Rubinov A, Beiran I, Krasnitz I, Miller B. Bilateral optic neuritis after inactivated influenza vaccination. The Israel Medical Association journal : IMAJ. 2012;14(11):705-7

81. Dadeya S, Guliani BP, Gupta VS, Malik KPS, Jain DC. Retrobulbar neuritis following rabies vaccination. Tropical doctor. 2004;34(3):174-5

82. Lapphra K, Huh L, Scheifele DW. Adverse neurologic reactions after both doses of pandemic H1N1 influenza vaccine with optic neuritis and demyelination. Pediatric Infectious Disease Journal. 2011;30(1):84-6.10.1097/INF.0b013e3181f11126: 10.1097/INF.0b013e3181f11126

83. Chang H, Lee HL, Yeo M, Kim JS, Shin DI, Lee SS, et al. Recurrent optic neuritis and neuromyelitis optica-IgG following first and second human papillomavirus vaccinations. Clinical neurology and neurosurgery. 2016;144:126-8.10.1016/j.clineuro.2016.03.023: 10.1016/j.clineuro.2016.03.023

84. Esmanhotto BB, Rocha IM, Vilar CRL, Haluch RF, Valença TP. Neuromyelitis optica associated with yellow fever vaccination. Acta neurologica Belgica. 2021;121(2):567-9.10.1007/s13760-020-01396-1: 10.1007/s13760-020-01396-1

85. Menge T, Cree B, Saleh A, Waterboer T, Berthele A, Kalluri SR, et al. Neuromyelitis optica following human papillomavirus vaccination. Neurology. 2012;79(3):285-7.10.1212/WNL.0b013e31825fdead: 10.1212/WNL.0b013e31825fdead

86. Cho JH, Park Y, Woo N. A case of neuromyelitis optica spectrum disorder following seasonal influenza vaccination. Multiple sclerosis and related disorders. 2019;30:110-3.10.1016/j.msard.2019.01.052: 10.1016/j.msard.2019.01.052

87. Heekin R, Gandhy C, Robertson D. Seronegative neuromyelitis optica spectrum disorder following exposure to hepatitis b vaccination. Case Reports in Neurology. 2015;7(1):78-83.10.1159/000381826: 10.1159/000381826

88. Younes S, Sigireddi RR, Raviskanthan S, Mortensen PW, Lee AG. Bilateral myelin oligodendrocyte glycoprotein-related optic neuritis presenting after herpes zoster subunit vaccination. Canadian Journal of Ophthalmology. 2021;56(5):e157-e60.10.1016/j.jcjo.2021.02.036: 10.1016/j.jcjo.2021.02.036

89. Otiv M, Botre A, Shah P. Measles–rubella vaccine–associated MOG-antibody positive acute demyelinating encephalomyelitis with optic neuritis in a child. Therapeutic Advances in Vaccines and Immunotherapy. 2022;10.10.1177/25151355221115016: 10.1177/25151355221115016

90. Landa G, Marcovich A, Leiba H, Springer A, Bukelman A, Pollack A. Multiple branch retinal arteriolar occlusions associated with smallpox vaccination. Journal of Infection. 2006;52(1):e7-e9.10.1016/j.jinf.2005.04.019: 10.1016/j.jinf.2005.04.019

91. Shah P, Zaveri JS, Haddock LJ. Acute macular neuroretinopathy following the administration of an influenza vaccination. Ophthalmic Surgery Lasers and Imaging Retina. 2018;49(10):e165-e8.10.3928/23258160-20181002-23: 10.3928/23258160-20181002-23

92. Liu JC, Nesper PL, Fawzi AA, Gill MK. Acute macular neuroretinopathy associated with influenza vaccination with decreased flow at the deep capillary plexus on OCT angiography. American Journal of Ophthalmology Case Reports. 2018;10:96-100.10.1016/j.ajoc.2018.02.008: 10.1016/j.ajoc.2018.02.008

93. Juncal VR, Bansal A, Hamli H, Muni RH. Paracentral acute middle maculopathy following hepatitis B vaccine. American Journal of Ophthalmology Case Reports. 2022;25.10.1016/j.ajoc.2022.101422: 10.1016/j.ajoc.2022.101422

94. Kwok T, Al-Bermani A. Two rare cases of retinal vasculitis following vaccination. Scottish medical journal. 2013;58(2):e10-e2.10.1177/0036933013482660: 10.1177/0036933013482660

95. Moysidis SN, Koulisis N, Patel VR, Kashani AH, Rao NA, Humayun MS, et al. The second blind spot: Small retinal vessel vasculopathy after vaccination against Neisseria meningitidis and yellow fever. Retinal Cases and Brief Reports. 2017;11:S18-S23.10.1097/ICB.0000000000000391: 10.1097/ICB.0000000000000391

96. Jabbour S, Shekhawat NS, Chen A, Woreta FA. Presumed Herpes Zoster Ophthalmicus Reactivation Following Recombinant Zoster Vaccination. Cornea. 2021;40(2):248-50.10.1097/ICO.0000000000002537: 10.1097/ICO.0000000000002537

97. Nguyen LN, Parikh SU, Batliwala SY, Davis AS, Riaz KM. Temporal profile and treatment of purpureocillium lilacinum keratitis secondary to herpes zoster reactivation following influenza vaccination. International Medical Case Reports Journal. 2020;13:455-9.10.2147/IMCRJ.S265724: 10.2147/IMCRJ.S265724

98. Lu TJ, Ta CN. Reactivation of Herpes Zoster Keratitis Following Shingrix Vaccine. Case Reports in Ophthalmology. 2022;13(1):104-8.10.1159/000522272: 10.1159/000522272

99. Jastrzebski A, Brownstein S, Ziai S, Saleh S, Lam K, Jackson WB. Reactivation of herpes zoster keratitis with corneal perforation after zoster vaccination. Cornea. 2017;36(6):740-2.10.1097/ICO.0000000000001203: 10.1097/ICO.0000000000001203

100. Lehmann A, Matoba A. Reactivation of Herpes Zoster Stromal Keratitis After HZ/su Adjuvanted Herpes Zoster Subunit Vaccine. Ophthalmology. 2018;125(11):1682.10.1016/j.ophtha.2018.08.030: 10.1016/j.ophtha.2018.08.030

101. Khalifa YM, Jacoby RM, Margolis TP. Exacerbation of zoster interstitial keratitis after zoster vaccination in an adult. Archives of Ophthalmology. 2010;128(8):1079-80.10.1001/archophthalmol.2010.142: 10.1001/archophthalmol.2010.142

102. Krall P, Kubal A. Herpes zoster stromal keratitis after varicella vaccine booster in a pediatric patient. Cornea. 2014;33(9):988-9.10.1097/ICO.0000000000000199: 10.1097/ICO.0000000000000199

103. Jenkins R, Chiew YF, Langford D, Johns P, Huang S, Miller N. A case of conjunctivitis associated with oral poliovirus type 2 vaccine (OPV). Pathology. 2004;36(5):505-6.10.1080/00313020412331282735: 10.1080/00313020412331282735

104. Kitaichi N, Ariga T, Ohno S, Shimizu T. Acute unilateral conjunctivitis after rubella vaccination: The detection of the rubella genome in the inflamed conjunctiva by reverse transcriptase- polymerase-chain reaction [7]. British Journal of Ophthalmology. 2006;90(11):1436-7.10.1136/bjo.2006.096008: 10.1136/bjo.2006.096008

105. Nazir HI, Hess AA, Soni A, Potter KA. A Case of Leukocytoclastic Vasculitis and Associated Conjunctivitis Following MMR Vaccine Administration. Case Reports in Dermatological Medicine. 2023;2023.10.1155/2023/9001287: 10.1155/2023/9001287
